# Revealing potential lipid biomarkers in clear cell renal cell carcinoma using targeted quantitative lipidomics

**DOI:** 10.1186/s12944-021-01572-z

**Published:** 2021-11-13

**Authors:** Wen Li, Xiaobin Wang, Xianbin Zhang, Peng Gong, Degang Ding, Ning Wang, Zhifeng Wang

**Affiliations:** 1 Carson International Cancer Centre, Shenzhen University General Hospital and Shenzhen University Clinical Medical Academy Centre, Shenzhen University, 1098 Xueyuan Road, Shenzhen, 518000 Guangdong China; 2 Key Laboratory of Optoelectronic Devices and Systems, College of Physics and Optoelectronic Engineering, Shenzhen University, Shenzhen, 518060 China; 3 Health Science Center, School of Medicine, Shenzhen University, Shenzhen, 518060 China; 4 Department of General Surgery, Shenzhen University General Hospital, Xueyuan Road 1098, Shenzhen, 518055 China; 5 Department of Urology, Henan Provincial People’s Hospital, Zhengzhou University People’s Hospital, Henan University People’s Hospital, No. 7 Weiwu Road, Zhengzhou City, 450003 Henan Province China

**Keywords:** Clear cell renal cell carcinoma, Lipids, Lipidomics, Lipid metabolite, Lipid biomarker, Lipid quantification, UPLC-MS/MS, Differentially expressed lipids

## Abstract

**Background:**

The high drug resistance and metabolic reprogramming of clear cell renal cell carcinoma (ccRCC) are considered responsible for poor prognosis. In-depth research at multiple levels is urgently warranted to illustrate the lipid composition, distribution, and metabolic pathways of clinical ccRCC specimens.

**Methods:**

In this project, a leading-edge targeted quantitative lipidomic study was conducted using 10 pairs of cancerous and adjacent normal tissues obtained from ccRCC patients. Accurate lipid quantification was performed according to a linear equation calculated using internal standards. Qualitative and quantitative analyses of lipids were performed with multiple reaction monitoring analysis based on ultra-performance liquid chromatography (UPLC) and mass spectrometry (MS). Additionally, a multivariate statistical analysis was performed using data obtained on lipids.

**Results:**

A total of 28 lipid classes were identified. Among them, the most abundant were triacylglycerol (TG), diacylglycerol (DG), phosphatidylcholine (PC), and phosphatidylethanolamine (PE). Cholesteryl ester (CE) was the lipid exhibiting the most considerable difference between normal samples and tumor samples. Lipid content, chain length, and chain unsaturation of acylcarnitine (CAR), CE, and DG were found to be significantly increased. Based on screening for variable importance in projection scores ≥1, as well as fold change limits between 0.5 and 2, 160 differentially expressed lipids were identified. CE was found to be the most significantly upregulated lipid, while TG was observed to be the most significantly downregulated lipid.

**Conclusion:**

Based on the absolute quantitative analysis of lipids in ccRCC specimens, it was observed that the content and change trends varied in different lipid classes. Upregulation of CAR, CE, and DG was observed, and analysis of changes in the distribution helped clarify the causes of lipid accumulation in ccRCC and possible carcinogenic molecular mechanisms. The results and methods described herein provide a comprehensive analysis of ccRCC lipid metabolism and lay a theoretical foundation for cancer treatment.

**Supplementary Information:**

The online version contains supplementary material available at 10.1186/s12944-021-01572-z.

## Background

According to cancer statistics reported in 2021, renal cell carcinoma ranks sixth among new cancer cases in men and ranks ninth among new cases in women [1]. Clear cell renal cell carcinoma (ccRCC) accounts for 70% of all renal cell carcinoma patients and is the main pathological feature of lipid accumulation [2]. Loss of the Von Hippel Lindau (VHL) gene and deletion of a part of chromosome 3p are involved in the initial steps, and vascular endothelial growth factor, PI3K, mTOR, and carbonic anhydrase IX have been defined as therapeutic targets [3]. Owing to poor prognosis attributable to drug resistance and immune escape, it is suggested that the discovery of more potential molecular mechanisms holds considerable promise [4, 5].

Metabolomics based on nuclear magnetic resonance (NMR), chromatography, and mass spectrometry (MS) can be considered to systematically analyze the variations under different physiological conditions using a combination of genomics and proteomics [6]. As an independent branch of metabolomics, lipidomics helps to comprehensively and systematically identify and quantify lipids to reveal key drivers of disease pathology. The lipid metabolites and pathways strategy (LIPID MAPS) consortium proposed a lipid classification system and divided lipids into the following eight categories: (1) fatty acyls (FAs); (2) glycerolipids (GL); (3) glycerophospholipids (GP), including phosphatidylcholine (PC), phosphatidylserine (PS), phosphatidylethanolamine (PE), phosphatidylinositol (PI), phosphatidic acid (PA), and cardiolipin (CL); (4) sphingolipids (SL), including ceramides (Cer), sphingomyelin (SM), and sphingosine (SPH); (5) prenol lipids (PR); (6) sterol lipids (ST); (7) saccharolipids; and (8) polyketides [7–9]. Apart from acting as constituents of biological structural components and participating in signal transduction, lipids also bind proteins to enable expansion of the metabolic regulatory network [10].

The diversity of lipid structures and the complexity of analytical methods are bottlenecks in systematic studies. With the development of high-throughput and high-precision technologies that rely on liquid chromatography and tandem MS, research on lipid function and metabolic regulation has advanced to the stage of omics. Targeted quantitative lipidomics aids detection of the precise content of specific substances to help provide diversified data for the potential discovery of biomarkers and drug targets [11, 12].

## Methods

### Study participants

All 10 pairs of tumor tissues and adjacent normal tissues were obtained from the Urology Department, Henan Provincial People’s Hospital. The study was approved by the Medical Ethics Committee of Henan Provincial People’s Hospital (no. 2019074) and was conducted in accordance with the Declaration of Helsinki. None of the patients involved in the study presented with any major underlying disease (Table 1).

**Table 1:** Clinical information of 10 patients

Characteristics	Value N (%) or Mean ± SD
Age	54.00 ± 10.40
Sex (Male)	10 (80%)
T1/T2 States	7 / 3
Tumor diameter (cm)	5.03 ± 2.40
Hypertension	10 (10%)
Cigarette smoking	10 (10%)
ALT, U/L	20.92 ± 14.40
AST, U/L	21.58 ± 7.49
TP, g/L	64.07 ± 10.10
TBIL, μmol/L	10.60 ± 6.08
ALP, U/L	72.37 ± 22.34
GGT, U/L	26.01 ± 13.68
TBA, μmol/L	1.94 ± 1.21
CHE, KU/L	7.94 ± 2.85
LDH, U/L	215.38 ± 42.83
GLDH-D, U/L	3.89 ± 1.75
NEFA-D, mmol/L	0.28 ± 0.17
CHOL, mmol/L	5.10 ± 0.64
TG, mmol/L	1.83 ± 0.23
HDL-C, mmol/L	1.11 ± 0.13
LDL-C, mmol/L	3.36 ± 0.53
APO-A1, g/L	1.12 ± 0.23
APOB100, g/L	1.07 ± 0.20
LPa, mg/dL	4.40 ± 1.98
UREA, mmol/L	5.78 ± 1.49
CREA, μmol/L	103.50 ± 39.03
UA, μmol/L	339.33 ± 74.27
GLU, mmol/L	5.78 ± 1.18

ALT: alanine aminotransferase; AST: aspartate aminotransferase; TP: total protein; TBIL: total bilirubin; ALP: alkaline phosphatase; GGT: glutamyl transferase; TBA: total bile acid; CHE: cholinesterase; LDH: lactate dehydrogenase; GLDH-D: glutamate dehydrogenase; NEFA-D: non-estesterified fatty acid; CHOL: cholesterol; TG: triglycerides; HDL-C: high density lipoprotein; LDL-C: low density lipoprotein; APO-A1: Apolipoprotein-A1; APOB100: Apolipoprotein B100; LPa: lipoprotein; CREA: Creatinine; UA: Uric acid; GLU: Glucose

### Sample preparation and extraction

The samples were measured by weight (20 mg each) and were then added to 1 mL lipid extract (methyl tert-butyl ether/methanol = 3/1, v/v, mixed with the internal standard). The internal standards were listed in Table 2. With the addition of steel balls, the mixtures were homogenized using a ball mill. After subjection to vortexing for 2 min and after sonication for 5 min, 200 μL of water was added. The samples were centrifuged for 10 min at 12000 rpm. The supernatant was aspirated and concentrated, and reconstituted with 200 μL of phase B (acetonitrile/isopropanol (10/90, v/v) with 0.1% formic acid and 10 mmol/L ammonium formate).

**Table 2:** The internal standard used in this study

Glass	Abbreviations	internal standards	Concentration (nmol/mL, μM)
Free fatty acid	FFA	FFA(18:2)-d11	0.2
Acylcarnitine	CAR	CAR(16:0)-d3	0.2
Eicosanoids	Eicosanoid	5S-HETE-d8	0.04
Cholesterol	Cho	Cho-d7	5
Cholesteryl ester	CE	CE(18:1)-d7	2
Bile Acid	BA	GCDCA-d4	0.04
Sphingosine	SPH	SPH(18:1)-d7	0.04
Ceramide	Cer	Cer(d18:1(d7)/18:0)	0.2
Ceramide 1-phosphates	CerP	CerP(d18:1/8:0)	0.4
Hexosylceramide	HexCer	HexCer(d18:1(d5)/18:0)	0.4
Sphingomyelin	SM	SM(d18:1(d9)/15:0)	0.2
Diacylglycerol	DG	DG(17:0/17:0)_d5	0.2
Triacylglycerol	TG	TG(17:0/17:1/17:0)_d5	0.2
Lysophophatidylcholine	LPC	LPC(16:0)-d31	0.2
alkyl-Lysophophatidylcholine	LPC-O	LPC(16:0)-d31	0.2
Lysophosphatidylethanolamine	LPE	LPE(14:0)	0.2
alkenyl-Lysophosphatidylethanolamine	LPE-P	LPE(14:0)	0.2
Lysophosphatidylglycerol	LPG	LPG(14:0)	0.2
Lysophosphatidylinositol	LPI	LPI(17:1)	0.2
Lysophosphatidylserine	LPS	LPS(17:1)	0.2
Phosphatidylcholine	PC	PC(16:0(d31)/18:1)	0.2
alkyl-glycerophosphocholines	PC-O	PC(16:0(d31)/18:1)	0.2
Phosphatidylethanolamine	PE	PE(16:0(d31)/18:1)	0.2
alkenyl-glycerophosphoethanolamines	PE-P	PE(16:0(d31)/18:1)	0.2
Phosphatidylglycerol	PG	PG(16:0(d31)/18:1)	0.4
Phosphatidylinositol	PI	PI(16:0(d31)/18:1)	0.2
Phosphatidylserine	PS	PS(16:0(d31)/18:1)	0.4
Coenzyme Q	CoQ	CoQ10-d9	0.4

### Ultra-performance liquid chromatography (UPLC) conditions

The ExionLC™ AD (AB Sciex, Framingham, USA) UPLC instrument was used by considering conditions as follows: (1) chromatographic column: the Thermo Accucore C30 column (2.6 μm, 100 mm × 2.1 mm ID); (2) mobile phase: phase A, acetonitrile/water (60/40, v/v, with 0.1% formic acid and 10 mmol/L ammonium formate); phase B, acetonitrile/isopropanol (10/90, v/v, with 0.1% formic acid and 10 mmol/L ammonium formate); (3) gradient washing program: 80/20 (phase A/B, v/v) at 0 min, 70/30 (phase A/B, v/v) at 2 min, 40/60 (phase A/B, v/v) at 4 min, 15/85 (phase A/B, v/v) at 9 min, 10/90 (phase A/B, v/v) at 14 min, 5/95 (phase A/B, v/v) at 15.5 min, 5/95 (phase A/B, v/v) at 17.3 min, 80/20 (phase A/B, v/v) at 17.5 min, 80/20 (phase A/B, v/v) at 20 min; (4) the flow rate was 0.35 mL/min, the column temperature was 45 °C, and the injection volume was 2 μL.

### Electrospray ionization-MS/MS conditions

The QTRAP® 6500+ (AB Sciex, Framingham, USA) mass spectrometer was used. The electrospray ionization source temperature was 500 °C. The MS voltage values were 5500 V (positive ion mode) and − 4500 V (negative ion mode). The pressure of ion source gas 1 was 45 psi, while that of ion source gas 2 was 55 psi; the pressure of the curtain gas was 35 psi. Each ion pair was scanned using optimal declustering potential and collision energy settings in the triple quadrupole.

### Data preprocessing

The MetWare Database (MWDB) was constructed based on the information available on standard products. Quantification was performed in the multiple reaction monitoring (MRM) mode. Only specified ions were collected. The chromatographic peaks of all targets were integrated, and quantitative analysis was performed using the internal standard.

A part of each sample was mixed to prepare a quality control, which was then analyzed using the total ion current and MRM metabolite detection multimodal graph (extracted ion current). MS data were processed using the Analyst 1.6.3 software based on the local lipid database. The MS peaks detected in different samples for each substance were calibrated to ensure accurate quantification.

### Lipid quantitative analysis

Different concentrations (0.2 nmol/L, 0.5 nmol/L, 1 nmol/L, 2 nmol/L, 5 nmol/L, 10 nmol/L, 20 nmol/L, 50 nmol/L, 100 nmol/L, 200 nmol/L, 500 nmol/L, 1000 nmol/L, 2000 nmol/L, 5000 nmol/L, and 10,000 nmol/L) of standard solutions were prepared using a mixture of dichloromethane/methanol to obtain data on the peak intensity and the corresponding quantitative signals. The chromatographic peak area indicates the relative content of the corresponding substance; this information was substituted into the linear equation and calculation formula (Table 3-4). The following calculation formula was subjected to unit conversion, and the sample content could be obtained by directly substituting the corresponding values in the generated standard curves.

$$ \mathrm{X}=\left(0.001\times \mathrm{c}\times \mathrm{V}\times \mathrm{v}1\right)/\mathrm{v}2/\mathrm{m} $$

**Table 3:** Standard curve linear equation

Class	Equation	r	LLOQ	ULOQ
BA	y = 5.89032 x + 0.00349	0.99952	0.001	1
CAR	y = 10.63664 x - 9.00378e-4	0.99178	0.005	5
CE	y = 4.15246 x - 0.00124	0.99310	0.01	5
Cer	y = 2.90771 x + 0.00126	0.99628	0.002	2
CerP	y = 0.14978 x + 7.83392e-5	0.99815	0.005	2
Cho	y = 4.27736 x + 0.02240	0.99768	0.005	5
CoQ	y = 3.64824 x + 1.73992	0.99749	0.002	2
DG	y = 7.54751 x + 0.04692	0.99412	0.002	2
Eicosanoid	y = 61.65750 x + 0.08742	0.99661	0.001	1
FFA	y = 22.05858 x - 0.21591	0.99297	0.02	10
HexCer	y = 1.40675 x - 2.86002e-5	0.99152	0.002	2
LPC	y = 2.87925 x - 0.00565	0.99009	0.01	5
LPC-O	y = 2.87925 x - 0.00565	0.99009	0.01	5
LPE	y = 0.43538 x - 0.00138	0.99562	0.02	5
LPE-P	y = 0.43538 x - 0.00138	0.99562	0.02	5
LPG	y = 0.62714 x - 1.52689e-5	0.99848	0.005	2
LPI	y = 0.06717 x + 0.00722	0.99669	0.002	2
LPS	y = 0.10692 x + 2.42176e-5	0.99127	0.01	2
PC	y = 1.39542 x - 6.09826e-4	0.99651	0.002	10
PC-O	y = 1.39542 x - 6.09826e-4	0.99651	0.002	10
PE	y = 14.19927 x - 0.02853	0.99327	0.01	5
PE-P	y = 1.01408 x - 0.00815	0.99613	0.02	5
PG	y = 6.97289 x + 0.01424	0.99033	0.01	10
PI	y = 3.05988 x - 0.15491	0.99717	0.02	5
PS	y = 6.52668 x - 0.37092	0.99437	0.01	10
SM	y = 0.68841 x - 5.22709e-4	0.99110	0.002	10
SPH	y = 1.80406 x + 4.28477e-4	0.99513	0.005	2
TG	y = 1.33356 x + 5.11722e-4	0.99125	0.005	10

Class: lipid classification; Equation: linear equation; r: the correlation coefficient; LLOQ (nmol/mL): lower limit of quantification; ULOQ (nmol/mL): upper limit of quantification

**Table 4:** The specific lipid specie that was used to generate the calibration curve

Class	Lipid name	CAS number	Supplier	Article number
FFA	FA(18:2)	60–33-3	sigma-Aldrich	L1376
CAR	CAR(16:0)	2364-67-2	Supelco	91,503
Eicosanoid	5S-HETE	73,307–52-5	cayman	34,210
Cho	cholesterol	57–88-5	sigma-Aldrich	C3045
CE	CE(18:2)	604–33-1	sigma-Aldrich	C0289
BA	Glycochenodeoxycholic acid	16,564–43-5	sigma-Aldrich	G0759
SPH	SPH(18:1)	213–78-4	Avanti	
Cer	Cer(d18:1/17:0)	67,492–21-4	Avanti	860517P
CerP	CerP(d18:1/16:0)	2,146,303–22-9	Avanti	860533P
HexCer	HexCer(d18:1/16:0)	2,260,795–77-3	Cayman	26,009
SM	SM(d18:1/17:0)	121,999–64-2	Avanti	860585P
DG	DG(17:0/17:0)	98,896–81-2	Cayman	26,942
TG	TG(17:0/17:0/17:0)	2438-40-6	sigma-Aldrich	T2151
LPC	LPC(17:0)	50,930–23-9	Avanti	855676P
LPC-O	LPC(17:0)	50,930–23-9	Avanti	855676P
LPE	LPE(18:0)	69,747–55-3	Avanti	856715P
LPE-P	LPE(18:0)	69,747–55-3	Avanti	856715P
LPG	LPG(18:0)	326,495–23-2	Avanti	858214P
LPI	LPI(18:1)	799,268–53–4	Avanti	850149P
LPS	LPS(18:1)	326,589–90-6	Avanti	858143P
PC	PC(17:0/17:0)	70,897–27-7	Avanti	850360P
PC-O	PC(17:0/17:0)	70,897–27-7	Avanti	850360P
PE	PE(17:0/17:0)	140,219–78-9	Avanti	830756P
PE-P	PE(P-18:0/18:1)	144,371–68-6	Avanti	852758P
PG	PG(17:0/17:0)	799,268–52-3	Avanti	830456P
PI	PS(16:0/18:1)	321,863–21-2	Avanti	840034P
PS	PI(16:0/18:1)	50,730–13-7	Avanti	850142P
CoQ	CoQ10	303–98-0	Supelco	7386

X: the content of lipid in the sample (nmol/g);

c: the concentration value obtained by substituting the integrated peak area ratio in the sample into the standard curve (nmol/mL);

V: volume of the reconstituted solution (μL);

v1: volume of the sample extraction solution (μL);

v2: volume of the collected supernatant (μL); and.

m: sample mass (g).

## Results

### Lipid composition analysis

**Fig. 1:**
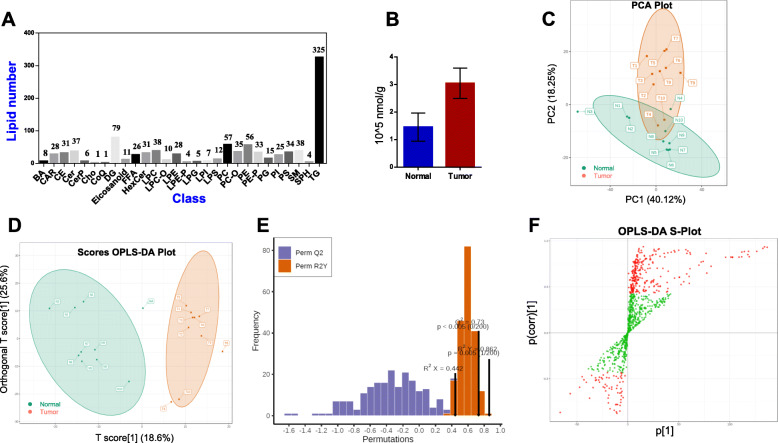
Lipid identification. **A** The type and number of identified lipids. **B** The total content of lipid molecules. **C** The PCA analysis on tumor and normal samples. **D** OPLS-DA score chart. The abscissa represents the predicted principal component and abscissa direction indicates the gap between groups. The ordinate represents the orthogonal principal component and the ordinate direction indicates the gap within the group. **E** The verification diagram OPLS-DA model. R2X = 0.442, R2Y = 0.862, Q2 = 0.73, *P* < 0.005. **F** S-plot of OPLS-DA model. The abscissa represents the covariance between the principal component and the lipid, and the ordinate represents the correlation coefficient between the principal component and the lipid. The red points indicate that the VIP value of these lipids is greater than or equal to 1, and the green points indicate that the VIP value of these lipids is less than 1

Qualitative analysis was performed after the completion of lipid extraction and MWDB establishment. The linear equations and correlation coefficients of the standard curves are presented in Table 2-4. The calculation formula was further introduced to obtain information on the absolute content. A total of 28 lipid subclasses, and their corresponding compounds, were detected (Fig. 1A). The lipid subclass presenting with the highest number of compounds was triacylglycerol (TG). The total content of quantified lipids was calculated by considering the sum of the contents of all lipid compounds in the same sample, and value of the summation was significantly higher in tumor tissues than that estimated in normal tissues (Fig. 1B).

Principal component analysis (PCA) is a common and unsupervised pattern-recognition multi-dimensional statistical analysis that is used to transform potentially correlated variables into linearly uncorrelated variables through orthogonal transformation [13]. Partial least squares-discriminant analysis (PLS-DA) is a multivariate statistical analysis with supervised pattern recognition that helps maximize the distinction between groups and aids the discovery of different metabolites. Orthogonal partial least squares discriminant analysis (OPLS-DA) is used to connect orthogonal signal correction with PLS-DA to generate score maps of each group to further highlight the differences [14]. PCA analysis was performed to determine the separation trend between the groups (Fig. 1C). The OPLS-DA model was established by using the OPLSR. Anal function in the MetaboAnalyst R software package to compare the degree of variability between the groups and between the samples within the group (Fig. 1D). R^2^X and R^2^Y represent the interpretation rates of the X and Y matrices, respectively. The predictive ability is represented by Q^2^. The evaluation shows that the OPLS-DA model is ideal and stable (Fig. 1E-F).

### Differences in lipid subclass content

**Fig. 2:**
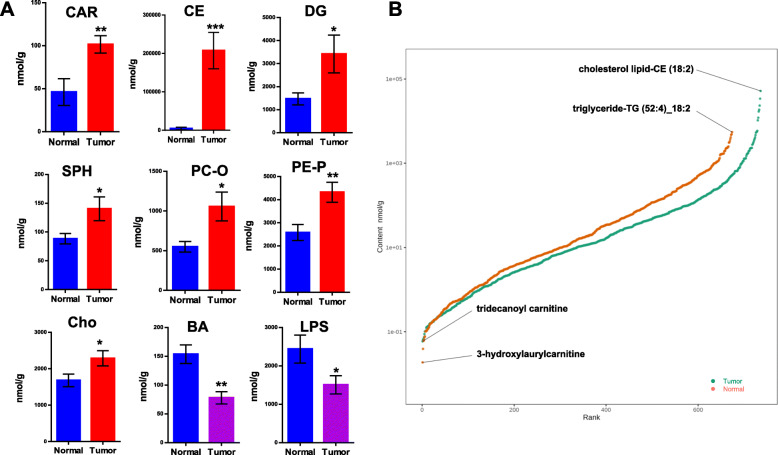
Changes in lipid subclass content. **A** The up- and down-regulated lipid subtypes between groups. **B** Dynamic distribution of lipid content. Each point represents a lipid molecule. The ordinate represents the corresponding content of each lipid molecule, and the lipid molecules with the lowest and highest content are marked

Functional research on lipids is mainly conducted and expressed in units of subclasses. Different lipid subclasses demonstrate evident differences in their biological functions. Acylcarnitine (CAR), cholesteryl ester (CE), diacylglycerol (DG), SPH, alkylglycerophosphocholines (PC-O), alkenylglycerophosphoethanolamines (PE-P), and cholesterol (Cho) presented with significantly higher levels in tumors than those observed in normal samples. Additionally, the content of bile acid (BA) and lysophosphatidylserine (LPS) in tumor tissues was lower than that in normal tissue (Fig. 2A). There was no significant difference in the content of other types of lipids (Fig. S1).

Analysis of the dynamic distribution range of lipid content revealed the lipid molecules presenting with the lowest and highest levels, as well as highlighted changes in the span of lipid content. In normal samples, 3-hydroxylaurylcarnitine exhibited the lowest levels and triglyceride-TG (52:4)_18:2 exhibited the highest levels. In tumor tissues, the lipid with the lowest content was identified as tridecanoyl carnitine, and the lipid with the highest content was identified as cholesterol lipid-CE (18:2) (Fig. 2B).

### Lipid chain length analysis

The length of the lipid chain is defined as the sum of the carbon atoms in the fatty acid chain of the lipid molecule. In addition to the lipid content, chain length is closely related to lipid function. The chain length can affect the thickness of the plasma membrane, which in turn affects the fluidity of the cell membrane, the activity and function of the relevant lipid transport protein, and the target protein [15].

**Fig. 3:**
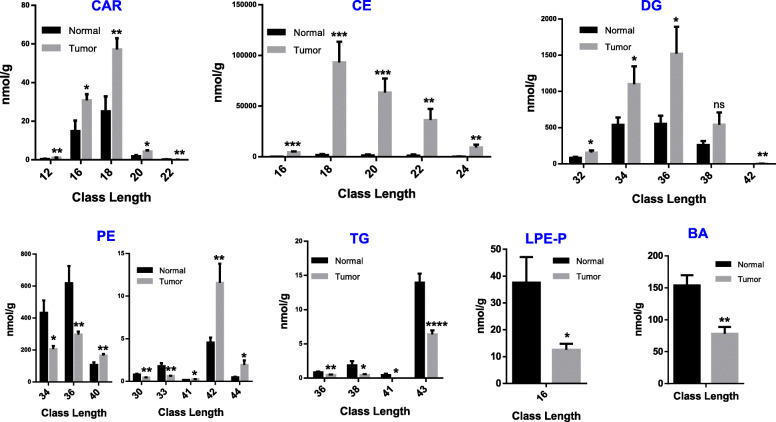
The carbon chain length analysis. The content of lipid compounds corresponding to different carbon chain lengths were listed in each group

The data on the levels of lipid compounds presenting with the same chain length were added together, and the differences in the presence of different chain lengths were noted. Compared with the normal group, the content of CAR in the tumor group was found to be increased significantly at chain lengths of 12, 16, 18, and 20, but was reduced by a chain length of 22. For all chain lengths, the CE content observed in the tumor group was significantly higher than that noted in the normal group. The content of DG in the tumor group increased with chain lengths of 32, 34, 36, and 42, and there was no significant difference between other chain lengths. Increased content of PE in chain lengths of 40, 41, 42, and 44, and decreased in chain lengths of 30, 33, 34, and 36, were observed in tumor samples. In tumors, lipid contents were decreased in TG with lengths of 36, 38, 41, and 43, as well as in alkenyl-lysophosphatidylethanolamine (LPE-P) and BA (Fig. 3). The chains in the other subclasses between groups were measured and the significant differences are marked with an asterisk (Fig. S2).

### Lipid chain unsaturation analysis

The degree of unsaturation of the lipid chain is defined as the sum of the number of double bonds in the fatty acid chain of the lipid molecule. The saturation of cell membrane lipids affects the fluidity of the membrane, which in turn affects the proliferation and invasiveness of tumor cells [16].

**Fig. 4:**
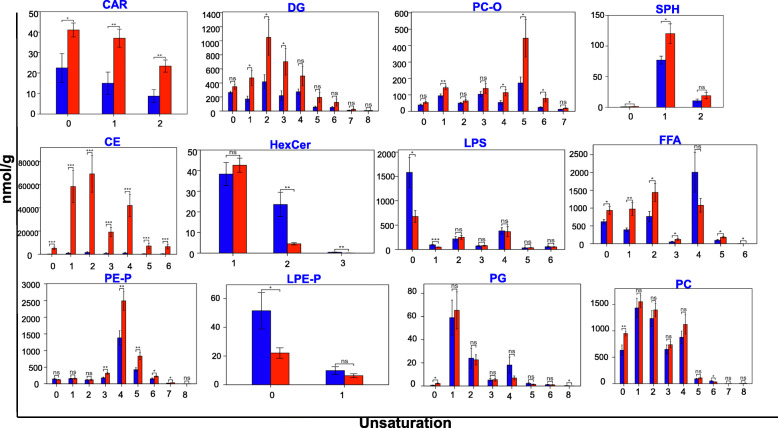
The chain unsaturation analysis. The content of lipid compounds corresponding to different carbon chain saturations in each group were listed

The data on the levels of lipid compounds with the same number of unsaturated bonds were added. Compared with the normal group, it was observed that the lipid content of all unsaturated bonds in CAR and CE increased significantly in the tumor group. The content of lipid compounds with a partial number of unsaturated bonds in other lipid types showed a significant increase or decrease in tumors (Fig. 4, Fig. S3). Lipid chain length and unsaturation level affect the mechanical properties of the respective biological macromolecules. Studies have found that a gradual accumulation of specific long-chain fatty acids (LCFAs) in CD8+ T cells in the pancreas not only disrupts mitochondrial function, but also promotes the TCA cycle through the β oxidation process of FA [17]. The types of LCFAs and lipids with different saturations may provide clues for the maintenance of tumor progression and immune cell metabolic reprogramming in the tumor microenvironment [17].

### Screening of differentially expressed lipids

Differential lipids were screened based on the fold change (FC) and variable importance in projection (VIP) values. Compared with the normal group, the differences were considered significant in the tumor groups with FC ≥ 2 and FC ≤ 0.5, respectively. Lipids with VIP ≥ 1 were regarded as significantly different.

**Fig. 5:**
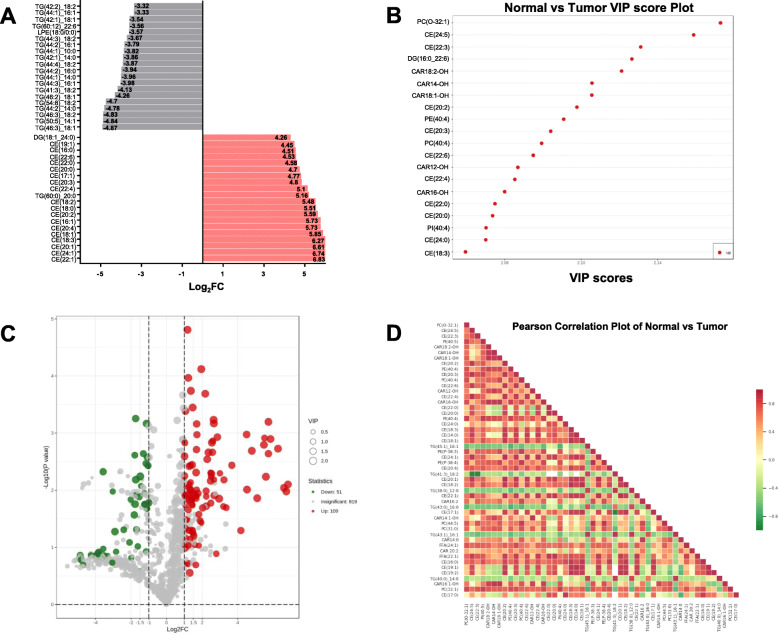
Differentially expressed lipids. **A** Bar chart of differentially expressed lipids. FC: Fold change. **B** The top 20 lipids ranked by VIP value of differentially expressed lipids. **C** The volcano map of differentially expressed lipids. Each point in the volcano map represents a lipid. Significantly up-regulated lipids are represented by red dots, and significantly down-regulated lipids are represented by green dots. The size of the dot represents the VIP value. **D** Correlation analysis on the significantly different lipids. Different colors represent the level of Pearson’s correlation coefficient

The FC in each group was compared and calculated according to their binary logarithm. The top 20 upregulated and downregulated lipids were ranked according to a VIP ≥ 1. The lipids with the most upregulated expression were CE (22:1) (FC = 6.83), CE (24:1) (FC = 6.74), and CE (20:1) (FC = 6.61). Most lipids among the top 20 upregulated lipids were identified as cholesteryl ester, except for TG (60:0)_20:0 FC = 5.16) and DG (18:1_24:0) (FC = 4.26). Additionally, the lipids with most downregulated expression were TG (46:3)_18:1 (FC = 4.87), TG (50:5)_14:1 (FC = 4.84), and TG (46:3)_18:2 (FC = 4.83). Most lipids among the top 20 downregulated lipids were TG, except for lysophosphatidylethanolamine (LPE) (18:0/0:0) (FC = 3.57) (Fig. 5A, Supplementary Table 1). The top 20 lipids with the greatest VIP value in each group in the OPLS-DA model were selected, and expression levels of all were found to be upregulated (Fig. 5B, Supplementary Table 2).

A total of 109 upregulated lipids and 51 downregulated lipids are indicated in red and green, respectively, in the volcano plot (Fig. 5C). Each point represents a metabolite. The closeness of significantly different lipids was measured via differential lipid correlation analysis to further understand their mutual adjustment relationship. Pearson correlation analysis was performed for the significantly different lipids. The top 50 differential lipids with the greatest VIP values were selected (Fig. 5D). Interestingly, there is a more obviously negative correlation between several types of TG with other lipids.

## Discussion

Accurate identification and absolute quantification of lipids are important for the comprehensive study of lipid metabolism. Fatty acid oxidation (FAO) is essential for tumor metabolic reprogramming, a mechanism which provides the necessary energy and biological intermediate products [18]. The upregulated FAO in tumors contributes to tumor growth and progression, and to the development of the malignant phenotype characterized by aggressive, metastatic, and drug resistance traits [19–24]. In the β-oxidation process, FAs are activated and degraded after binding to coenzyme A (CoA) in the cytoplasm. Inhibition of β-oxidation decreases FA metabolism, preventing hydroperoxide formation and ferroptosis in a manner dependent on glutathione or glutathione peroxidase in ccRCC [25]. Carnitine palmitoyltransferase 1 (CPT1) catalyzes the transfer of long-chain fatty acyl-CoA into CAR, which is then carried by the carnitine translocator (CAT) across the inner mitochondrial membrane. CPT2 catalyzes the conversion of long-chain CAR into long-chain acyl-CoA. High expression of CPT1 has been reported in breast cancer [26], lung cancer [27], gastric cancer [28], prostate cancer [29], ovarian cancer [30], nasopharyngeal cancer [31] and chronic lymphoblastic leukemia [32]. CPT1 is considered the rate-limiting enzyme for FAO, and the level of CAR reflects the degree of FAO to a certain extent [33]. Cholesterol is not only an important component of the membrane structure, but also serves as a precursor for BAs, sterol hormones, vitamins, and oxidized cholesterol [34]. Tumor cells hijack and store excess cholesterol contents in LD in the form of CE to provide energy, to promote tumor growth, and to increase metastasis of prostate and pancreatic tumors [35, 36]. Blockade of cholesterol intake via inactivation of the liver X receptors causes the death of malignant brain cancer cells, resulting in a positive therapeutic effect [37]. DG is produced by the phosphatase enzyme reaction through phosphatidic acid in the endoplasmic reticulum or via the decomposition of TG during lipolysis [38]. As a secondary messenger, DG activates a signal cascade reaction to promote tumor growth [39]. The quantitative lipidomic results in this study showing an accumulation of CAR, CE, and DG, which can be mutually supported by the conclusion of the increase of upstream metabolic enzymes in these literatures. In addition to the detection of peripheral blood content, the study of these metabolite levels in tumors is conducive to the in-depth study of metabolic reprogramming driven by endogenous and exogenous factors and is valuable for the exploration of combined therapy related to the immune regulation of the ccRCC tumor microenvironment.

After combining with cell membranes to release arachidonic acid, fat-soluble BA promotes reactive oxygen species (ROS) production and induces DNA damage [40]. The functions of BA in mediating inflammation, in promoting proliferation, and in inhibiting apoptosis proceed by a series of signal transductions [41]. Intestinal bacteria modify BAs that enter the intestine, promoting their disintegration. The resulting metabolites promote the differentiation of anti-inflammatory Treg cells and inhibit the proliferation of pro-inflammatory Th17 cells, thereby regulating the immune response [42, 43]. The content of BA in ccRCC and the regulation of tumor growth and immune cells have not been studied thus far. The significantly lower BA content reported in the present study, and its function in tumor tissues, merit further confirmation.

### Comparisons with other studies and what does the current work add to the existing knowledge

Apart from exhibition of genetic mutations and epigenetic regulation [44], ccRCC is a metabolic disease that involves metabolic reprogramming during tumorigenesis and progression [45]. Lipid accumulation in ccRCC is related to the absorption and excretion of lipid metabolites [46, 47]. The role of considerable amounts of lipid droplets (LDs) accumulated in ccRCC remains controversial [48]. Abnormal hypoxia inducible factor (HIF) expression caused by VHL gene deletion has been shown to promote tumor angiogenesis, glycolysis, and metastasis [49]. Different isoforms of phospholipid-binding protein AnxA3 help modulate LD storage in ccRCC cell lines [47]. The number of LDs is closely related to the concentration of glucose in the cells. Fatty acid synthesis is vigorous and, under conditions when glucose concentration is sufficient, TG is synthesized and stored in LD [50]. The metabolite lactic acid under anaerobic conditions is used as an energy carrier for mutual transmission within and between tissues in lung and pancreatic cancers [51]. There is a need to utilize highly advanced metabolic research methods to explore the increased FA uptake and synthesis, glycolysis enzyme expression, glycolysis paradox (Warburg effect), pentose phosphate pathway, uptake of external glutamine and arginine, and the decreased β-oxidation of FA and oxidative phosphorylation [52].

Combined analysis of renal cancer proteomics and non-targeted metabolomics has helped reveal grade-dependent metabolic reprogramming [33, 53]. The detection of metabolites using NMR illustrates an overall signature in the urine samples of patients [54, 55]. An HIF-1 targeted gene, NADH dehydrogenase (ubiquinone) 1 alpha subcomplex 4-like 2 (NDUFA4L2), was overexpressed in ccRCC tumor samples and was found to promote proliferation, migration, and drug resistance, based on a combination of untargeted metabolomic and transcriptomic analyses [56]. MS-dependent untargeted lipidomic research has further helped identify the enrichment of polyunsaturated FAs (PUFAs) based on the analysis of long-chain FAs (LCFAs) [57]. Compared to the existing studies, the present study revealed quantitative detection of a variety of lipid metabolites in ccRCC at one time and provided more comprehensive data support for ccRCC progression based on more detailed lipid profile information.

### Study strengths and limitations

Lipid signaling molecules participate in proliferation, death signaling, and tumor metabolism [10]. Lipidomic research is conducted by including differential lipid screening, regulation of lipid function, and lipid network construction. Non-targeted lipidomics is unbiased and systematically reflects the lipid characteristics of living bodies. The repeatability of non-targeted lipidomics is poor and the linear range is limited. Targeted quantitative lipidomic research, as performed in the present study, benefits from wide coverage, high throughput, reproducibility, sensitivity, and accuracy. It is, however, a biased analysis based on specific types of lipids; hence, exogenous lipids are necessary for consideration as internal standards. The existing research methods implemented may destroy the physiological structure of lipids during sample extraction and preparation; this may cause significant differences in the results. Thus, it is imperative to modify the extraction steps and to standardize database construction for different types of biological specimens included and analyzed.

## Conclusions

Tumor cells establish their own malignant proliferation through metabolic reprogramming, which is an important feature that distinguishes them from normal cells. A comprehensive and in-depth characterization of tumor metabolic characteristics provides an opportunity to identify and develop potential tumor diagnosis and treatment targets. Global lipidomic analysis with approaches that change dynamically over time will be used to analyze tumor pathogenesis with greater accuracy. In this project, 28 subclasses of lipids were detected using the UPLC-MS/MS detection platform. Different lipid changes in ccRCC tumor tissues, such as increased CAR, CE, DG, and SPH levels, and a decrease in BA and LPS, may be related to an increase in the components constituting the membrane structure required for cell proliferation and the rearrangement of events in energy metabolism; this aspect warrants further in-depth verification.

## Supplementary Information

**Additional file 1: Fig. S1.** The non-significant lipid count between groups. There are no significant differences in all group comparisons. **Fig. S2.** Chain length of lipid subtypes between groups. Significant differences are marked with an asterisk, and no significant differences are not marked. **Fig. S3** Chain unsaturation of lipid subtypes between groups. Significant differences are marked with an asterisk.

**Additional file 2: Supplementary Table 1.** Differentially expressed lipids.

**Additional file 3: Supplementary Table 2.** VIP value.

## Data Availability

All data included in this study are available upon request by contact with the corresponding author.

## Abbreviations

ccRCC: clear cell renal cell carcinoma
MRM: Multiple Reaction Monitoring
UPLC: Ultra-Performance Liquid Chromatography
MS: Mass Spectrometry
TG: Triacylglycerol
DG: Diacylglycerol
PC: Phosphatidylcholine
PE: Phosphatidylethanolamine
CE: Cholesteryl ester
CAR: Acylcarnitine
VIP: Variable Importance in Projection
VHL: von Hippel Lindau
NMR: Nuclear Magnetic Resonance
FA: Fatty acyls
PUFA: polyunsaturated FA
LCFA: long chain FA
NDUFA4L2: NADH dehydrogenase (ubiquinone) 1 alpha subcomplex 4-like 2
GP: Glycerophospholipids
PS: Phosphatidylserine
PI: Phosphatidylinositol
PA: Phosphatidic acid
CL: Cardiolipin
SL: Sphingolipids
Cer: Ceramides
SM: Sphingomyelin
SPH: Sphingosine
ST: Sterol lipids
PR: Prenol lipids
PK: Polyketides
LD: lipid droplet
HIF: Hypoxia Inducible Factor
PCA: Principal Component Analysis
PLS-DA: Partial least squares-discriminant analysis
OPLS-DA: Orthogonal partial least squares discriminant analysis
PC-O: alkylglycerophosphocholines
PE-P: alkenylglycerophosphoethanolamines
Cho: Cholesterol
BA: Bile acid
LPS: Lysophosphatidylserine
LPE-P: alkenyl-Lysophosphatidylethanolamine
LPE: Lysophosphatidylethanolamine
FC: Fold change
DA: Differential Abundance
FAO: Fatty acid oxidation
ROS: Reactive oxygen species
CPT1: Carnitine palmitoyltransferase 1
CAT: Carnitine translocator
ALT: alanine aminotransferase
AST: aspartate aminotransferase
TP: total protein
TBIL: total bilirubin
ALP: alkaline phosphatase
GGT: glutamyl transferase
TBA: total bile acid
CHE: cholinesterase
LDH: lactate dehydrogenase
GLDH-D: glutamate dehydrogenase
NEFA-D: non-estesterified fatty acid
CHOL: cholesterol
TG: triglycerides
HDL-C: high density lipoprotein
LDL-C: low density lipoprotein
APO-A1: Apolipoprotein-A1
APOB100: Apolipoprotein B100
LPa: lipoprotein
CREA: Creatinine
UA: Uric acid
GLU: Glucose
