# Supplementary figures and images for: Revealing potential lipid biomarkers in clear cell renal cell carcinoma using targeted quantitative lipidomics
Source: Lipids Health Dis. 2021 Nov 13;20:160. doi: 10.1186/s12944-021-01572-z (PMC8590225; doi:10.1186/s12944-021-01572-z)

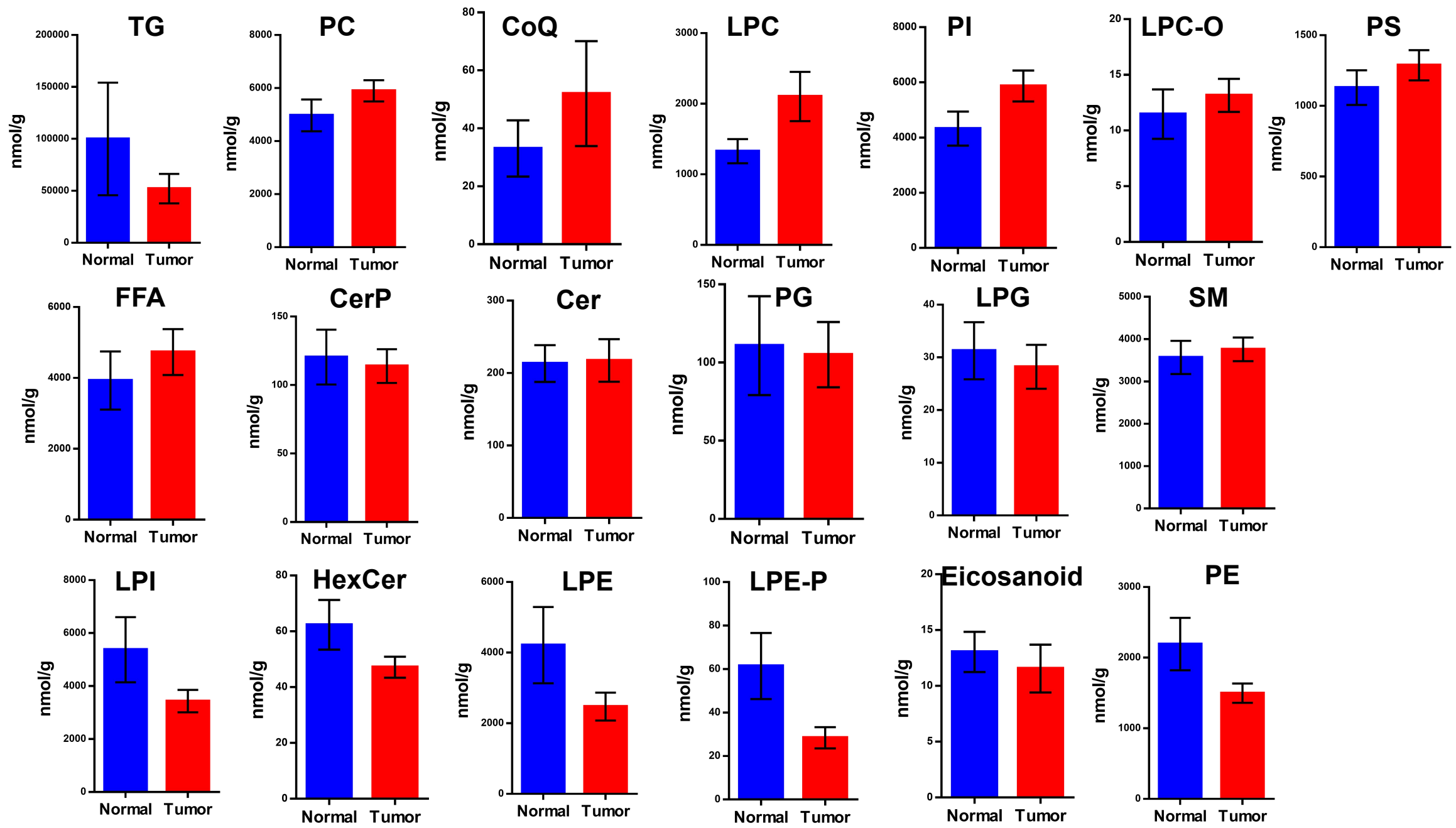

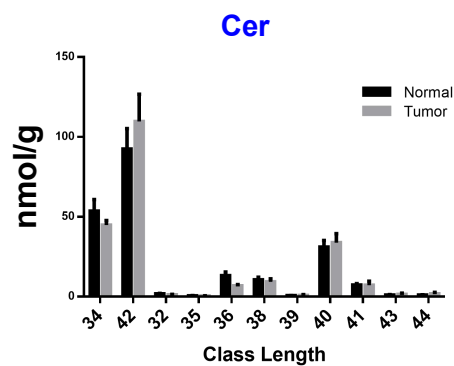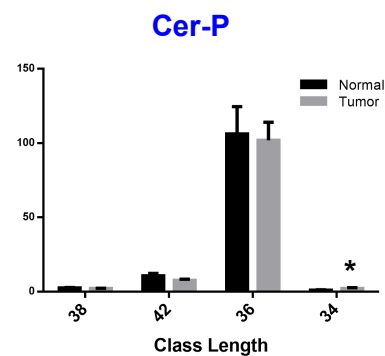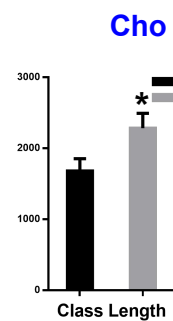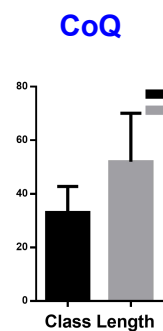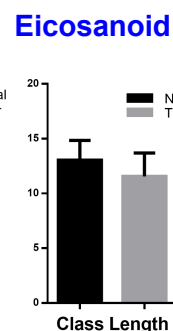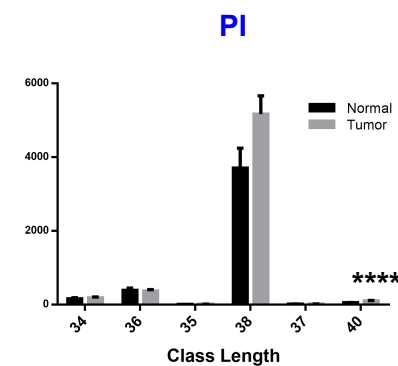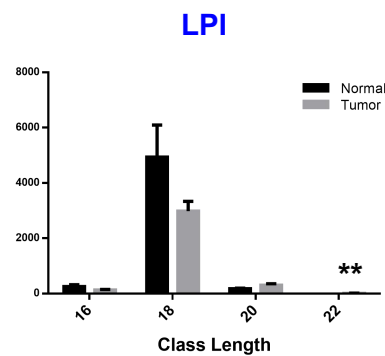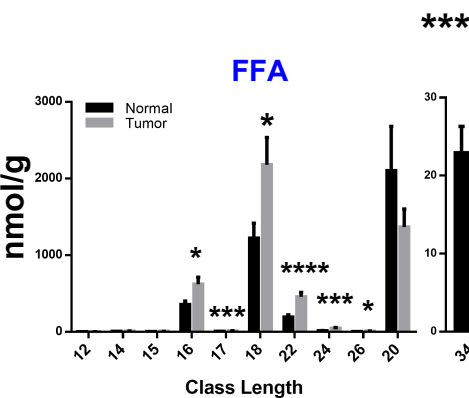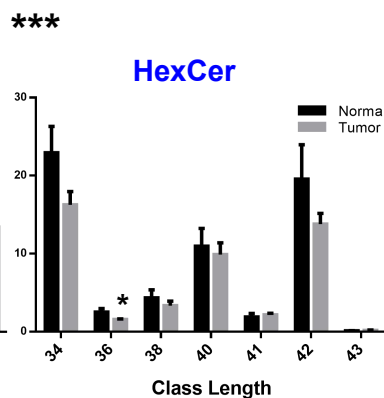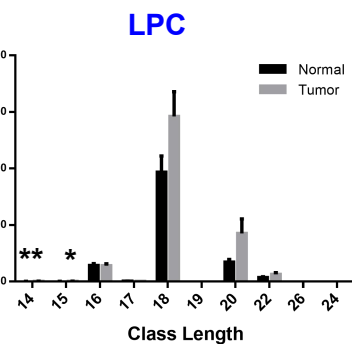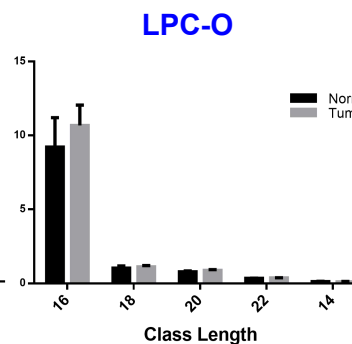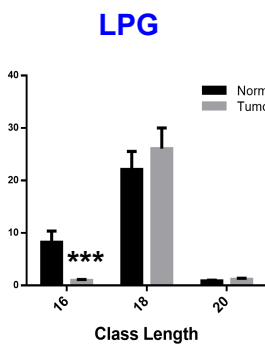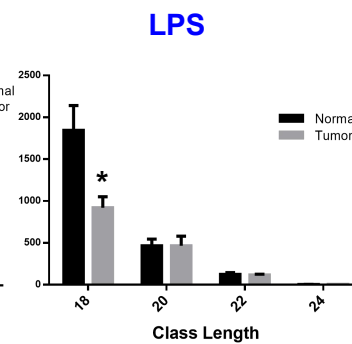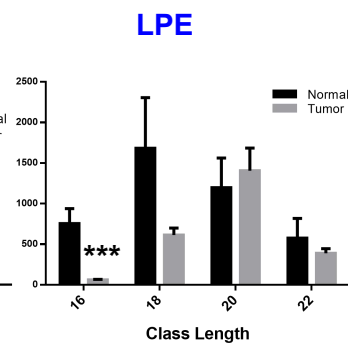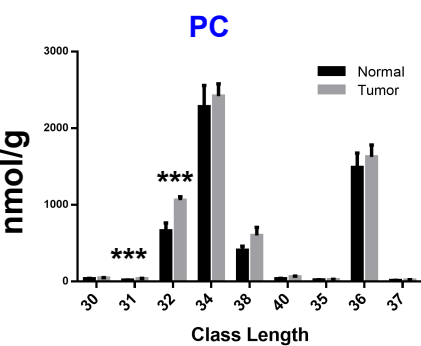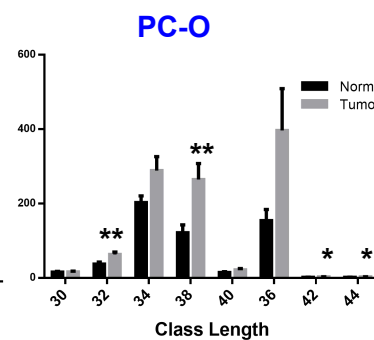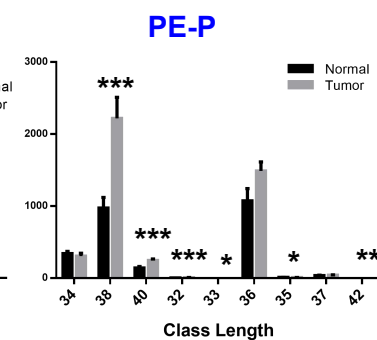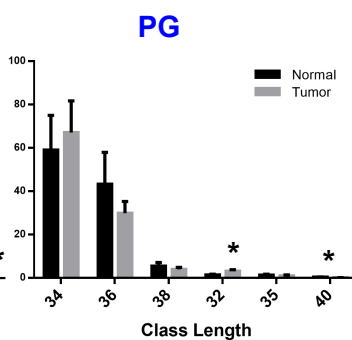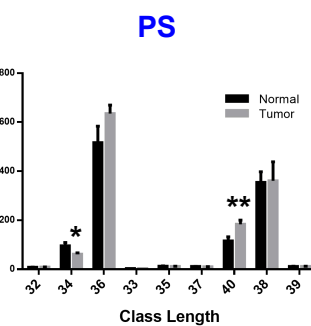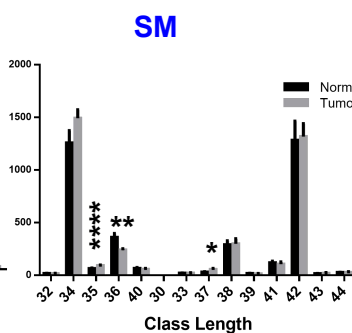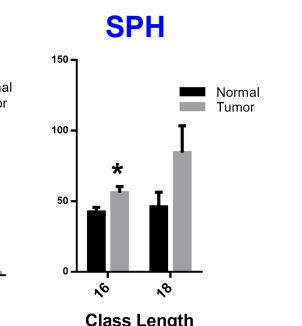

nmol/g

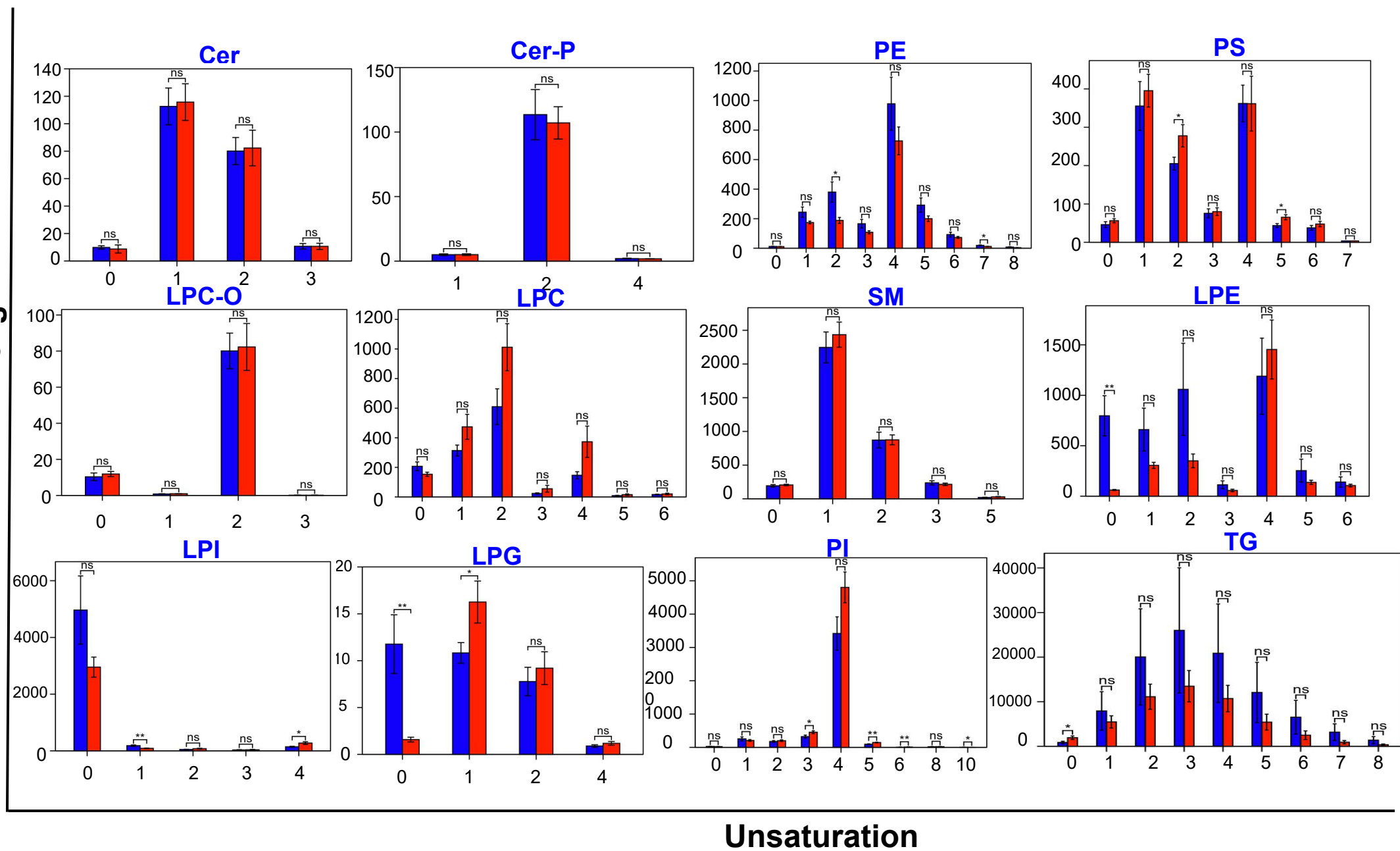

Supplement: Supplementary file 1 — Additional file 1: Fig. S1. The non-significant lipid count between groups. There are no significant differences in all group comparisons. Fig. S2. Chain length of lipid subtypes between groups. Significant differences are marked with an asterisk, and no significant differences are not marked. Fig. S3 Chain unsaturation of lipid subtypes between groups. Significant differences are marked with an asterisk. [file 12944_2021_1572_MOESM1_ESM.pdf]
