# Supplementary material for: Revealing potential lipid biomarkers in clear cell renal cell carcinoma using targeted quantitative lipidomics
Source: Lipids Health Dis. 2021 Nov 13;20:160. doi: 10.1186/s12944-021-01572-z (PMC8590225; doi:10.1186/s12944-021-01572-z)
Supplement: Supplementary file 2 — Additional file 2: Supplementary Table 1. Differentially expressed lipids. [file 12944_2021_1572_MOESM2_ESM.pdf]

Index	Formula	Compounds	Class I	Class II	lipidmaps ID	N1	N2	N3	N4	N5	N6	N7	N8	N9	N10	T1	T2	T3	T4	T5	T6	T7	T8	T9	T10	VIP	Pvalue	Fold_Change	Log2FC	Type
Lipid-Q-P-0047	C49H86O2	CE(22:1)	ST	CE	LMST01020025	1.16E+00	1.79E+00	1.03E+01	4.65E+02	9.55E-01	1.67E+00	2.21E+00	3.98E+00	1.17E+00	2.92E+00	4.61E+03	3.81E+03	3.80E+03	5.70E+00	1.26E+04	1.18E+04	1.34E+04	5.29E+03	7.89E+02	1.08E+01	1.98371897	0.007889931600162284	114.0235	6.8332	up
Lipid-Q-P-0048	C51H90O2	CE(24:1)	ST	CE	LMST01020020	1.02E+00	1.54E+00	6.95E+00	4.79E+02	1.59E+00	2.37E+00	2.55E+00	5.98E+00	1.35E+00	6.89E+00	4.81E+03	3.32E+03	3.14E+03	7.18E+00	1.10E+04	1.04E+04	1.59E+04	4.32E+03	1.44E+03	1.26E+01	2.02429239	0.010448126773323366	106.6873	6.7372	up
Lipid-Q-P-0046	C47H82O2	CE(20:1)	ST	CE	LMST01020011	3.77E+00	4.45E+00	2.71E+01	9.91E+02	3.29E+00	6.10E+00	6.49E+00	1.24E+01	3.28E+00	1.24E+01	8.09E+03	7.37E+03	6.14E+03	1.69E+01	2.00E+04	2.13E+04	2.89E+04	1.02E+04	2.06E+03	3.78E+01	2.00383605	0.009043829388296768	97.4351	6.6064	up
Lipid-Q-P-0055	C45H74O2	CE(18:3)	ST	CE	LMST01020009	7.70E+00	4.92E+00	5.84E+00	4.23E+02	3.37E+01	3.42E+01	2.87E+01	5.38E+01	2.86E+01	6.99E+01	5.73E+03	6.92E+03	5.03E+03	3.39E+01	7.81E+03	1.04E+04	9.87E+03	6.59E+03	8.91E+02	5.73E+01	2.03948589	0.0018755193465759348	77.3007	6.2724	up
Lipid-Q-P-0044	C45H78O2	CE(18:1)	ST	CE	LMST01020003	2.02E+02	1.11E+02	1.64E+02	4.19E+03	1.75E+02	1.74E+02	1.73E+02	3.68E+02	1.35E+02	2.42E+02	4.38E+04	3.94E+04	3.87E+04	3.51E+02	4.66E+04	5.23E+04	6.63E+04	4.62E+04	7.43E+03	6.41E+02	2.0338542	0.0012718089427367477	57.6041	5.8481	up
Lipid-Q-P-0042	C43H74O2	CE(16:1)	ST	CE	LMST01020006	9.54E+00	N/A	N/A	1.96E+02	1.77E+01	1.19E+01	1.25E+01	1.56E+01	1.25E+01	2.42E+01	2.08E+03	1.96E+03	1.64E+03	2.36E+01	2.86E+03	3.45E+03	4.81E+03	2.04E+03	1.07E+03	2.14E+01	1.78434281	0.002295968479896078	53.2364	5.7343	up
Lipid-Q-P-0061	C47H76O2	CE(20:4)	ST	CE	LMST01020014	3.47E+02	1.66E+02	5.44E+01	2.29E+03	2.70E+02	2.38E+02	2.53E+02	2.33E+02	1.98E+02	3.45E+02	2.70E+04	3.92E+04	2.86E+04	3.64E+02	3.32E+04	3.54E+04	3.39E+04	1.29E+04	2.30E+04	2.39E+02	2.01775549	6.371298353783264e-4	53.2342	5.7343	up
Lipid-Q-P-0053	C47H80O2	CE(20:2)	ST	CE	LMST01020012	4.88E+00	7.40E+00	2.30E+01	3.28E+03	1.38E+01	2.42E+01	1.86E+01	6.74E+01	9.91E+00	6.28E+01	1.45E+04	1.28E+04	1.16E+04	7.85E+01	2.84E+04	2.89E+04	4.65E+04	2.06E+04	5.39E+03	2.54E+02	2.09776133	0.005784286599443673	48.1447	5.5893	up
Lipid-Q-P-0035	C45H80O2	CE(18:0)	ST	CE	LMST01020007	1.24E+01	7.04E+00	2.06E+01	2.61E+02	4.70E+00	4.26E+00	4.33E+00	6.85E+00	3.21E+00	N/A	2.70E+03	1.69E+03	2.21E+03	6.16E+00	2.95E+03	1.58E+03	2.72E+03	2.04E+03	5.70E+02	2.13E+01	1.38219471	0.0012224162024926089	45.7161	5.5146	up
Lipid-Q-P-0051	C45H76O2	CE(18:2)	ST	CE	LMST01020008	8.95E+02	3.11E+02	1.69E+02	4.28E+03	1.30E+03	1.09E+03	8.99E+02	9.79E+02	4.42E+02	1.29E+03	6.58E+04	5.78E+04	5.47E+04	1.13E+03	7.04E+04	7.82E+04	1.06E+05	7.64E+04	8.70E+03	1.34E+03	2.00351709	0.0016079211025999387	44.6404	5.4803	up
Lipid-Q-P-0761	C63H122O6	TG(60:0)_20:0	GL	TG	LMGL03010003	7.65E-04	N/A	N/A	N/A	N/A	N/A	N/A	N/A	N/A	N/A	2.38E-02	N/A	2.48E-03	N/A	4.94E-02	3.43E-02	5.34E-02	9.62E-03	1.82E-02	N/A	1.62771098	N/A	35.6788	5.157	up
Lipid-Q-P-0062	C49H80O2	CE(22:4)	ST	CE	LMST01020018	3.81E+00	6.48E+00	2.94E+01	5.24E+03	7.79E+00	9.46E+00	1.36E+01	4.28E+01	9.72E+00	6.08E+01	9.15E+03	1.84E+04	9.78E+03	8.72E+01	3.92E+04	3.94E+04	5.34E+04	5.77E+03	1.08E+04	8.40E+01	2.06527943	0.013729953207811549	34.3158	5.1008	up
Lipid-Q-P-0057	C47H78O2	CE(20:3)	ST	CE	LMST01020013	1.36E+01	5.20E+00	1.69E+01	3.87E+03	5.73E+01	5.23E+01	5.96E+01	1.03E+02	2.90E+01	1.23E+02	9.10E+03	8.81E+03	1.00E+04	1.63E+02	1.89E+04	1.98E+04	2.80E+04	1.27E+04	1.30E+04	1.33E+02	2.08404265	0.0020481957165235706	27.8476	4.7995	up
Lipid-Q-P-0043	C44H76O2	CE(17:1)	ST	CE	LMST01020023	N/A	N/A	N/A	3.12E+01	N/A	N/A	N/A	N/A	1.64E+00	N/A	5.18E+02	5.03E+02	4.43E+02	N/A	5.61E+02	5.13E+02	8.79E+02	4.09E+02	2.08E+02	2.38E+00	1.97311375	N/A	27.3232	4.7721	up
Lipid-Q-P-0037	C47H84O2	CE(20:0)	ST	CE	LMST01020010	N/A	N/A	N/A	2.76E+01	N/A	N/A	N/A	N/A	N/A	N/A	5.88E+02	2.10E+02	3.46E+02	N/A	1.97E+03	1.13E+03	1.63E+03	5.34E+02	4.56E+01	3.41E+00	2.0536364	N/A	25.968	4.6987	up
Lipid-Q-P-0038	C49H88O2	CE(22:0)	ST	CE	LMST01020016	N/A	N/A	N/A	1.13E+01	N/A	N/A	N/A	N/A	N/A	N/A	1.79E+02	6.77E+01	1.09E+02	N/A	8.86E+02	4.09E+02	5.96E+02	1.64E+02	2.67E+01	1.63E+00	2.05489912	N/A	23.9803	4.5838	up
Lipid-Q-P-0070	C49H76O2	CE(22:6)	ST	CE	LMST01020019	3.06E+01	1.84E+01	9.77E+00	2.26E+03	2.90E+01	2.34E+01	2.68E+01	3.06E+01	1.47E+01	1.04E+02	4.90E+03	5.88E+03	6.18E+03	7.64E+01	7.91E+03	8.97E+03	1.88E+04	2.47E+03	3.52E+03	7.82E+01	2.07491009	0.009698162525155596	23.0716	4.528	up
Lipid-Q-P-0033	C43H76O2	CE(16:0)	ST	CE	LMST01020005	9.94E+01	3.65E+01	4.79E+01	5.55E+02	3.83E+01	3.18E+01	3.46E+01	5.22E+01	2.90E+01	7.44E+01	3.26E+03	2.70E+03	3.07E+03	5.95E+01	4.06E+03	2.70E+03	3.75E+03	2.10E+03	9.41E+02	4.65E+01	1.92974996	0.0010603200583290979	22.7122	4.5054	up
Lipid-Q-P-0045	C46H80O2	CE(19:1)	ST	CE	-	N/A	N/A	N/A	2.32E+01	N/A	N/A	N/A	N/A	N/A	N/A	4.84E+02	4.50E+02	3.58E+02	N/A	5.94E+02	7.37E+02	8.68E+02	3.88E+02	1.66E+02	N/A	1.92000386	N/A	21.8174	4.4474	up
Lipid-Q-P-0185	C45H86O5	DG(18:1_24:0)	GL	DG	-	N/A	N/A	N/A	3.45E-01	5.28E-02	N/A	N/A	N/A	N/A	N/A	1.59E+00	1.28E+00	2.36E+00	N/A	7.24E+00	7.22E+00	6.84E+00	2.51E+00	1.52E+00	N/A	1.80017195	N/A	19.1925	4.2625	up
Lipid-Q-P-0823	C45H82O6	TG(42:2)_18:2	GL	TG	-	1.74E+01	2.27E+01	6.92E+01	8.82E-01	9.89E-01	9.75E-01	1.06E+00	8.20E-01	2.44E-01	1.43E+00	2.44E-01	1.97E-01	2.16E-01	2.11E-01	6.20E+00	1.11E-01	3.01E+00	1.73E-01	7.21E-02	N/A	1.24445783	0.16641882469729177	0.1001	-3.3205	down
Lipid-Q-P-0772	C47H88O6	TG(44:1)_16:1	GL	TG	LMGL03013322	6.53E+01	6.41E+01	1.95E+02	2.01E+00	2.48E+00	4.44E+00	3.69E+00	3.83E+00	3.12E+00	4.24E+00	5.38E+00	2.39E+00	4.01E+00	2.42E+00	8.68E+00	7.31E-01	3.84E+00	3.49E+00	1.07E+00	2.59E+00	1.42063326	0.14305125951181652	0.0994	-3.3306	down
Lipid-Q-P-0768	C45H84O6	TG(42:1)_18:1	GL	TG	-	2.68E+01	2.98E+01	8.06E+01	8.62E-01	1.33E+00	9.56E-01	9.57E-01	9.48E-01	6.21E-01	2.26E+00	4.81E-01	4.53E-01	3.44E-01	5.36E-01	6.57E+00	2.51E-01	3.27E+00	1.92E-01	4.47E-02	3.47E-01	1.46445177	0.13936895137170058	0.086	-3.5395	down
Lipid-Q-P-1167	C63H98O6	TG(60:12)_22:6	GL	TG	LMGL03012142	4.06E+00	6.29E+00	1.29E+01	1.92E+00	1.20E-01	2.20E-01	3.08E-01	3.07E-02	1.58E-01	N/A	5.59E-01	5.41E-01	3.86E-01	1.78E-01	1.19E-01	N/A	N/A	5.52E-03	9.69E-02	7.18E-02	1.13723669	0.10577384916157952	0.0846	-3.5632	down
Lipid-Q-P-0357	C23H48NO7P	LPE(18:0/0:0)	GP	LPE	LMGP02050001	1.74E+01	1.08E+01	9.22E+00	6.02E+01	6.90E+01	9.94E+01	1.11E+02	4.87E+01	2.73E+01	2.06E+01	1.38E+00	4.39E+00	1.96E+00	4.91E+00	6.13E+00	4.02E+00	4.94E+00	3.15E+00	3.23E+00	5.68E+00	1.76814557	0.00472181567817237	0.084	-3.5735	down
Lipid-Q-P-0896	C47H84O6	TG(44:3)_18:2	GL	TG	-	4.74E+01	5.63E+01	1.59E+02	8.48E-01	1.72E+00	1.15E+00	1.44E+00	8.66E-01	4.22E-01	1.40E+00	5.76E-01	3.96E-01	3.80E-01	2.40E-01	1.15E+01	2.08E-01	5.24E+00	5.88E-01	3.91E-02	N/A	1.1910947	0.15690414939968275	0.0786	-3.6693	down
Lipid-Q-P-0826	C47H86O6	TG(44:2)_16:1	GL	TG	LMGL03014140	4.03E+01	3.80E+01	1.29E+02	8.46E-01	2.16E+00	2.10E+00	2.29E+00	3.51E+00	1.22E+00	3.32E+00	9.92E-01	1.15E+00	8.04E-01	1.63E+00	5.45E+00	4.98E-01	2.48E+00	7.66E-01	8.43E-01	1.50E+00	1.57169429	0.1419596547228136	0.0722	-3.7919	down
Lipid-Q-P-0773	C47H88O6	TG(44:1)_10:0	GL	TG	-	9.36E+01	1.24E+02	2.49E+02	5.26E+00	2.85E+00	2.82E+00	3.33E+00	1.69E+00	9.77E-01	4.06E+00	3.20E+00	2.00E+00	2.47E+00	6.05E-01	1.49E+01	3.63E-01	9.17E+00	1.07E+00	1.91E-01	4.97E-01	1.33922246	0.12005445408596688	0.0708	-3.8201	down
Lipid-Q-P-0769	C45H84O6	TG(42:1)_14:0	GL	TG	LMGL03013212	1.51E+01	1.59E+01	5.49E+01	2.83E-01	9.42E-01	1.27E+00	1.46E+00	1.03E+00	7.67E-01	1.86E+00	4.85E-01	4.02E-01	3.25E-01	4.66E-01	2.04E+00	2.74E-01	1.15E+00	3.46E-01	3.59E-01	6.10E-01	1.64562141	0.14190715794103217	0.0691	-3.8552	down
Lipid-Q-P-0962	C47H82O6	TG(44:4)_18:2	GL	TG	-	9.27E+00	7.97E+00	2.98E+01	5.31E-01	1.47E+00	1.10E+00	1.14E+00	6.21E-01	1.65E-01	7.01E-01	6.27E-02	7.72E-02	1.05E-01	3.28E-02	2.21E+00	3.33E-02	6.78E-01	3.53E-02	N/A	9.19E-03	1.64865581	0.1265550353586615	0.0684	-3.8699	down
Lipid-Q-P-0827	C47H86O6	TG(44:2)_16:0	GL	TG	-	1.26E+02	1.68E+02	4.44E+02	3.91E+00	2.92E+00	3.34E+00	4.01E+00	2.36E+00	1.37E+00	2.16E+00	2.23E+00	8.98E-01	1.07E+00	5.86E-01	2.95E+01	4.41E-01	1.24E+01	1.43E+00	2.93E-01	6.76E-01	1.38420486	0.15202087604389777	0.0653	-3.9368	down
Lipid-Q-P-0774	C47H88O6	TG(44:1)_14:0	GL	TG	LMGL03013217	1.08E+02	1.04E+02	2.98E+02	1.70E+00	1.87E+00	3.57E+00	4.07E+00	4.53E+00	2.16E+00	3.45E+00	3.29E+00	1.99E+00	3.29E+00	2.43E+00	1.25E+01	6.25E-01	4.96E+00	1.31E+00	1.13E+00	2.59E+00	1.39877266	0.13621933881902987	0.0642	-3.9613	down
Lipid-Q-P-0895	C47H84O6	TG(44:3)_16:1	GL	TG	LMGL03012816	1.43E+01	1.58E+01	4.52E+01	4.12E-01	5.20E-01	4.67E-01	7.22E-01	2.27E-01	2.14E-01	8.61E-01	3.14E-01	1.67E-01	1.60E-01	N/A	2.23E+00	8.06E-02	8.88E-01	6.76E-02	7.22E-02	N/A	1.18734171	0.1413568583629116	0.0632	-3.9839	down
Lipid-Q-P-0891	C44H78O6	TG(41:3)_18:2	GL	TG	-	9.60E-01	4.81E-01	1.93E+00	N/A	1.13E-01	2.48E-01	3.07E-01	1.96E-01	8.42E-02	N/A	N/A	N/A	N/A	N/A	N/A	N/A	3.08E-02	N/A	N/A	N/A	2.01102683	N/A	0.0571	-4.1304	down
Lipid-Q-P-0835	C49H90O6	TG(46:2)_18:1	GL	TG	LMGL03014145	2.74E+02	3.43E+02	8.04E+02	2.82E+00	4.01E+00	3.05E+00	3.45E+00	2.78E+00	1.77E+00	2.62E+00	6.81E+00	3.11E+00	5.78E+00	1.66E+00	3.72E+01	7.76E-01	1.44E+01	3.58E+00	1.05E+00	7.26E-01	1.13744574	0.13741267820831088	0.0521	-4.2626	down
Lipid-Q-P-1125	C57H94O6	TG(54:8)_18:2	GL	TG	LMGL03010471	9.63E+01	7.52E+01	4.32E+02	1.70E+00	9.21E-01	7.68E-01	1.27E+00	1.11E+00	4.15E-01	3.98E-01	4.54E+00	2.51E+00	3.98E+00	1.30E+00	6.71E+00	3.50E-02	1.87E+00	2.39E+00	9.42E-02	1.45E-01	1.12890069	0.20324889296896761	0.0386	-4.6953	down
Lipid-Q-P-0828	C47H86O6	TG(44:2)_14:0	GL	TG	LMGL03013218	3.94E+01	4.06E+01	1.56E+02	1.10E-01	7.38E-01	3.64E-01	7.61E-01	1.24E+00	6.18E-01	5.44E-01	4.78E-01	2.61E-01	4.82E-01	5.13E-01	3.62E+00	2.21E-01	1.50E+00	5.64E-01	2.06E-01	N/A	1.13435949	0.17042902843260999	0.0363	-4.7839	down
Lipid-Q-P-0901	C49H88O6	TG(46:3)_18:2	GL	TG	LMGL03013352	2.10E+02	2.40E+02	7.16E+02	1.57E+00	3.07E+00	2.15E+00	4.22E+00	2.65E+00	7.61E-01	2.67E+00	3.33E+00	1.48E+00	2.32E+00	7.30E-01	2.08E+01	3.35E-01	9.40E+00	2.08E+00	6.02E-01	5.90E-01	1.29989151	0.15040258556010255	0.0352	-4.8283	down
Lipid-Q-P-1023	C53H92O6	TG(50:5)_14:1	GL	TG	LMGL03012851	1.97E+02	1.79E+02	6.54E+02	6.57E-01	1.31E+00	8.50E-01	5.98E-01	1.04E+00	5.39E-01	3.24E-01	4.93E+00	2.00E+00	2.88E+00	6.20E-01	1.71E+01	1.51E-01	4.93E+00	3.35E+00	7.66E-02	1.29E-01	1.00703976	0.16420203693994925	0.0349	-4.8406	down
Lipid-Q-P-0902	C49H88O6	TG(46:3)_18:1	GL	TG	-	1.60E+02	1.76E+02	4.73E+02	2.18E+00	2.47E+00	2.30E+00	2.44E+00	1.93E+00	3.97E-01	3.38E+00	2.10E+00	5.95E-01	9.45E-01	5.19E-01	1.52E+01	3.92E-01	7.06E+00	9.56E-01	2.37E-01	2.08E-01	1.36385811	0.1360694531494935	0.0343	-4.8656	down
