# Supplementary material for: Revealing potential lipid biomarkers in clear cell renal cell carcinoma using targeted quantitative lipidomics
Source: Lipids Health Dis. 2021 Nov 13;20:160. doi: 10.1186/s12944-021-01572-z (PMC8590225; doi:10.1186/s12944-021-01572-z)
Supplement: Supplementary file 3 — Additional file 3: Supplementary Table 2. VIP value. [file 12944_2021_1572_MOESM3_ESM.pdf]

| Index | Formula | Compounds | Class I | Class II | lipidmaps ID | N1 | N2 | N3 | N4 | N5 | N6 | N7 | N8 | N9 | N10 | T1 | T2 | T3 | T4 | T5 | T6 | T7 | T8 | T9 | T10 | VIP | Pvalue | Fold_Change | Log2FC | Type |
|---|---|---|---|---|---|---|---|---|---|---|---|---|---|---|---|---|---|---|---|---|---|---|---|---|---|---|---|---|---|---|
| Lipid-Q-P-0483 | C40H80NO7P | PC(O-32:1) | GP | PC-O | LMGP01020182 | 4.05E+00 | 4.13E+00 | 1.62E+00 | 1.41E+01 | 3.60E+00 | 4.33E+00 | 4.61E+00 | 4.92E+00 | 5.56E+00 | 5.68E+00 | 1.64E+01 | 1.04E+01 | 2.16E+01 | 6.98E+00 | 2.24E+01 | 2.67E+01 | 2.81E+01 | 1.82E+01 | 3.05E+01 | 2.25E+01 | 2.17285453 | 7.602077661164569e-5 | 3.8729 | 1.9534 | up |
| Lipid-Q-P-0068 | C50H80O2 | CE(24:5) | ST | CE | - | N/A | N/A | N/A | 1.16E+03 | N/A | N/A | N/A | 3.22E+00 | N/A | 1.01E+01 | 1.38E+03 | 3.43E+03 | 1.65E+03 | 1.31E+01 | 5.51E+03 | 5.73E+03 | 7.81E+03 | 2.32E+03 | 2.41E+03 | 6.02E+00 | 2.15881614 | 0.013980756096266936 | 7.7598 | 2.956 | up |
| Lipid-Q-P-0058 | C49H82O2 | CE(22:3) | ST | CE | LMST01020042 | N/A | N/A | N/A | 2.09E+03 | N/A | N/A | N/A | 9.84E+00 | N/A | 1.64E+01 | 9.95E+02 | 2.08E+03 | 9.40E+02 | 1.78E+01 | 3.29E+03 | 2.15E+03 | 3.38E+03 | 1.05E+03 | 3.39E+03 | 2.08E+01 | 2.13111357 | 0.27889529750400427 | 2.4586 | 1.2978 | up |
| Lipid-Q-N-0239 | C49H85O13P | PI(40:5) | GP | PI | LMGP06010307 | 1.32E+01 | 1.47E+01 | 6.80E+00 | 3.26E+01 | 1.70E+01 | 1.44E+01 | 1.56E+01 | 2.10E+01 | 1.69E+01 | 3.16E+01 | 5.01E+01 | 3.06E+01 | 6.57E+01 | 2.41E+01 | 5.65E+01 | 4.39E+01 | 7.50E+01 | 4.43E+01 | 5.19E+01 | 3.18E+01 | 2.12650348 | 1.824249133491608e-4 | 2.5771 | 1.3657 | up |
| Lipid-Q-P-0028 | C25H45NO5 | CAR18:2-OH | FA | CAR | LMFA07070042 | 2.32E-02 | 3.45E-02 | 9.57E-03 | 1.28E+00 | 9.06E-02 | 7.06E-02 | 4.70E-02 | 2.26E-01 | 3.71E-01 | 6.89E-01 | 2.39E+00 | 1.70E+00 | 2.00E+00 | 3.57E-01 | 1.50E+00 | 1.43E+00 | 1.39E+00 | 3.38E+00 | 2.99E+00 | 6.75E-01 | 2.12108838 | 5.873594629947057e-4 | 6.267 | 2.6478 | up |
| Lipid-Q-P-0006 | C21H41NO5 | CAR14-OH | FA | CAR | LMFA07070045 | 1.44E-02 | 1.36E-02 | 6.55E-03 | 2.45E-01 | 1.91E-02 | 1.60E-02 | 1.02E-02 | 4.93E-02 | 7.57E-02 | 1.58E-01 | 5.08E-01 | 3.29E-01 | 6.09E-01 | 8.31E-02 | 1.83E-01 | 1.76E-01 | 2.94E-01 | 6.15E-01 | 6.06E-01 | 1.55E-01 | 2.10564694 | 0.0014692153015007565 | 5.8562 | 2.55 | up |
| Lipid-Q-P-0023 | C25H47NO5 | CAR18:1-OH | FA | CAR | LMFA07070025 | 3.81E-02 | 9.64E-02 | 1.64E-02 | 2.05E+00 | 2.16E-01 | 1.52E-01 | 1.01E-01 | 3.73E-01 | 9.24E-01 | 1.29E+00 | 4.20E+00 | 3.69E+00 | 3.58E+00 | 7.34E-01 | 2.19E+00 | 2.33E+00 | 2.42E+00 | 4.90E+00 | 7.12E+00 | 2.09E+00 | 2.10556587 | 6.795002158530279e-4 | 6.3212 | 2.6602 | up |
| Lipid-Q-P-0053 | C47H80O2 | CE(20:2) | ST | CE | LMST01020012 | 4.88E+00 | 7.40E+00 | 2.30E+01 | 3.28E+03 | 1.38E+01 | 2.42E+01 | 1.86E+01 | 6.74E+01 | 9.91E+00 | 6.28E+01 | 1.45E+04 | 1.28E+04 | 1.16E+04 | 7.85E+01 | 2.84E+04 | 2.89E+04 | 4.65E+04 | 2.06E+04 | 5.39E+03 | 2.54E+02 | 2.09776133 | 0.005784286599443673 | 48.1447 | 5.5893 | up |
| Lipid-Q-P-0554 | C45H82NO8P | PE(40:4) | GP | PE | LMGP02011200 | 1.51E+01 | 3.04E+01 | 6.80E+00 | 5.71E+01 | 2.31E+01 | 2.30E+01 | 2.51E+01 | 2.67E+01 | 2.19E+01 | 4.25E+01 | 5.88E+01 | 6.67E+01 | 6.76E+01 | 3.50E+01 | 6.18E+01 | 6.13E+01 | 5.91E+01 | 8.03E+01 | 7.02E+01 | 5.34E+01 | 2.09082398 | 1.5527347926853407e-5 | 2.2611 | 1.177 | up |
| Lipid-Q-P-0057 | C47H78O2 | CE(20:3) | ST | CE | LMST01020013 | 1.36E+01 | 5.20E+00 | 1.69E+01 | 3.87E+03 | 5.73E+01 | 5.23E+01 | 5.96E+01 | 1.03E+02 | 2.90E+01 | 1.23E+02 | 9.10E+03 | 8.81E+03 | 1.00E+04 | 1.63E+02 | 1.89E+04 | 1.98E+04 | 2.80E+04 | 1.27E+04 | 1.30E+04 | 1.33E+02 | 2.08404265 | 0.0020481957165235706 | 27.8476 | 4.7995 | up |
| Lipid-Q-P-0457 | C48H88NO8P | PC(40:4) | GP | PC | LMGP01010813 | 3.35E+00 | 6.48E+00 | 1.09E+00 | 1.60E+01 | 5.58E+00 | 4.85E+00 | 5.86E+00 | 5.48E+00 | 6.27E+00 | 9.60E+00 | 1.36E+01 | 1.83E+01 | 1.50E+01 | 1.30E+01 | 1.56E+01 | 1.70E+01 | 1.75E+01 | 1.50E+01 | 3.63E+01 | 1.72E+01 | 2.07930355 | 3.573938806210459e-4 | 2.7656 | 1.4676 | up |
| Lipid-Q-P-0070 | C49H76O2 | CE(22:6) | ST | CE | LMST01020019 | 3.06E+01 | 1.84E+01 | 9.77E+00 | 2.26E+03 | 2.90E+01 | 2.34E+01 | 2.68E+01 | 3.06E+01 | 1.47E+01 | 1.04E+02 | 4.90E+03 | 5.88E+03 | 6.18E+03 | 7.64E+01 | 7.91E+03 | 8.97E+03 | 1.88E+04 | 2.47E+03 | 3.52E+03 | 7.82E+01 | 2.07491009 | 0.009698162525155596 | 23.0716 | 4.528 | up |
| Lipid-Q-P-0004 | C19H37NO5 | CAR12-OH | FA | CAR | LMFA07070039 | 1.61E-02 | 1.47E-02 | 7.51E-03 | 2.57E-01 | 1.24E-02 | 9.51E-03 | 7.06E-03 | 4.13E-02 | 8.48E-02 | 1.85E-01 | 6.93E-01 | 3.20E-01 | 5.61E-01 | 8.11E-02 | 1.31E-01 | 1.83E-01 | 2.26E-01 | 3.99E-01 | 4.36E-01 | 2.21E-01 | 2.06687458 | 0.0021853022961139993 | 5.1213 | 2.3565 | up |
| Lipid-Q-P-0062 | C49H80O2 | CE(22:4) | ST | CE | LMST01020018 | 3.81E+00 | 6.48E+00 | 2.94E+01 | 5.24E+03 | 7.79E+00 | 9.46E+00 | 1.36E+01 | 4.28E+01 | 9.72E+00 | 6.08E+01 | 9.15E+03 | 1.84E+04 | 9.78E+03 | 8.72E+01 | 3.92E+04 | 3.94E+04 | 5.34E+04 | 5.77E+03 | 1.08E+04 | 8.40E+01 | 2.06527943 | 0.013729953207811549 | 34.3158 | 5.1008 | up |
| Lipid-Q-P-0009 | C23H45NO5 | CAR16-OH | FA | CAR | LMFA07070113 | 2.43E-02 | 8.83E-02 | 1.22E-02 | 1.83E+00 | 2.44E-01 | 1.91E-01 | 1.31E-01 | 3.56E-01 | 7.18E-01 | 1.34E+00 | 2.51E+00 | 1.92E+00 | 2.72E+00 | 7.23E-01 | 1.55E+00 | 2.52E+00 | 2.17E+00 | 3.12E+00 | 3.94E+00 | 1.12E+00 | 2.05995387 | 2.048482438175162e-4 | 4.5148 | 2.1747 | up |
| Lipid-Q-P-0038 | C49H88O2 | CE(22:0) | ST | CE | LMST01020016 | N/A | N/A | N/A | 1.13E+01 | N/A | N/A | N/A | N/A | N/A | N/A | 1.79E+02 | 6.77E+01 | 1.09E+02 | N/A | 8.86E+02 | 4.09E+02 | 5.96E+02 | 1.64E+02 | 2.67E+01 | 1.63E+00 | 2.05489912 | N/A | 23.9803 | 4.5838 | up |
| Lipid-Q-P-0037 | C47H84O2 | CE(20:0) | ST | CE | LMST01020010 | N/A | N/A | N/A | 2.76E+01 | N/A | N/A | N/A | N/A | N/A | N/A | 5.88E+02 | 2.10E+02 | 3.46E+02 | N/A | 1.97E+03 | 1.13E+03 | 1.63E+03 | 5.34E+02 | 4.56E+01 | 3.41E+00 | 2.0536364 | N/A | 25.968 | 4.6987 | up |
| Lipid-Q-N-0222 | C49H87O13P | PI(40:4) | GP | PI | LMGP06010853 | 7.36E+00 | 9.39E+00 | 5.64E+00 | 3.29E+01 | 1.73E+01 | 1.57E+01 | 1.69E+01 | 1.71E+01 | 1.40E+01 | 2.19E+01 | 4.40E+01 | 2.20E+01 | 4.64E+01 | 1.85E+01 | 3.66E+01 | 3.75E+01 | 4.61E+01 | 4.09E+01 | 4.85E+01 | 2.83E+01 | 2.0502295 | 1.0805313252581913e-4 | 2.3289 | 1.2196 | up |
| Lipid-Q-P-0039 | C51H92O2 | CE(24:0) | ST | CE | - | N/A | N/A | N/A | 1.38E+01 | N/A | N/A | N/A | N/A | N/A | N/A | 1.75E+02 | 7.67E+01 | 1.01E+02 | N/A | 2.92E+02 | 9.37E+01 | 1.46E+02 | 1.38E+02 | 3.99E+01 | 1.98E+00 | 2.050083 | N/A | 8.5449 | 3.0951 | up |
| Lipid-Q-P-0055 | C45H74O2 | CE(18:3) | ST | CE | LMST01020009 | 7.70E+00 | 4.92E+00 | 5.84E+00 | 4.23E+02 | 3.37E+01 | 3.42E+01 | 2.87E+01 | 5.38E+01 | 2.86E+01 | 6.99E+01 | 5.73E+03 | 6.92E+03 | 5.03E+03 | 3.39E+01 | 7.81E+03 | 1.04E+04 | 9.87E+03 | 6.59E+03 | 8.91E+02 | 5.73E+01 | 2.03948589 | 0.0018755193465759348 | 77.3007 | 6.2724 | up |
| Lipid-Q-P-0031 | C41H72O2 | CE(14:0) | ST | CE | LMST01020004 | N/A | N/A | N/A | 1.30E+01 | N/A | N/A | N/A | N/A | N/A | N/A | 9.45E+01 | 6.92E+01 | 8.10E+01 | 2.57E+00 | 1.21E+02 | 1.51E+02 | 1.86E+02 | 5.38E+01 | 4.29E+01 | N/A | 2.03687247 | N/A | 6.8659 | 2.7794 | up |
| Lipid-Q-P-0044 | C45H78O2 | CE(18:1) | ST | CE | LMST01020003 | 2.02E+02 | 1.11E+02 | 1.64E+02 | 4.19E+03 | 1.75E+02 | 1.74E+02 | 1.73E+02 | 3.68E+02 | 1.35E+02 | 2.42E+02 | 4.38E+04 | 3.94E+04 | 3.87E+04 | 3.51E+02 | 4.66E+04 | 5.23E+04 | 6.63E+04 | 4.62E+04 | 7.43E+03 | 6.41E+02 | 2.0338542 | 0.0012718089427367477 | 57.6041 | 5.8481 | up |
| Lipid-Q-P-0775 | C48H90O6 | TG(45:1)_16:1 | GL | TG | LMGL03014168 | 7.25E+00 | 8.19E+00 | 1.42E+01 | 2.15E+00 | 5.00E+00 | 4.80E+00 | 3.68E+00 | 7.30E+00 | 3.13E+00 | 5.31E+00 | 2.23E+00 | 2.90E+00 | 2.20E+00 | 2.89E+00 | 2.40E+00 | 1.02E+00 | 1.80E+00 | 1.76E+00 | 1.44E+00 | 3.19E+00 | 2.03074772 | 0.005691121223834251 | 0.358 | -1.482 | down |
| Lipid-Q-P-0595 | C43H80NO7P | PE(P-38:3) | GP | PE-P | LMGP02030055 | 1.67E+01 | 3.20E+01 | 7.56E+00 | 9.02E+01 | 3.33E+01 | 3.28E+01 | 3.22E+01 | 3.75E+01 | 4.57E+01 | 3.71E+01 | 1.20E+02 | 8.52E+01 | 1.07E+02 | 3.93E+01 | 1.17E+02 | 1.30E+02 | 1.34E+02 | 7.26E+01 | 2.86E+02 | 6.36E+01 | 2.02587115 | 0.004870883890849217 | 3.1632 | 1.6614 | up |
| Lipid-Q-P-0048 | C51H90O2 | CE(24:1) | ST | CE | LMST01020020 | 1.02E+00 | 1.54E+00 | 6.95E+00 | 4.79E+02 | 1.59E+00 | 2.37E+00 | 2.55E+00 | 5.98E+00 | 1.35E+00 | 6.89E+00 | 4.81E+03 | 3.32E+03 | 3.14E+03 | 7.18E+00 | 1.10E+04 | 1.04E+04 | 1.59E+04 | 4.32E+03 | 1.44E+03 | 1.26E+01 | 2.02429239 | 0.010448126773323366 | 106.6873 | 6.7372 | up |
| Lipid-Q-P-0600 | C43H78NO7P | PE(P-38:4) | GP | PE-P | LMGP02030003 | 2.19E+02 | 3.93E+02 | 9.82E+01 | 9.34E+02 | 3.85E+02 | 3.77E+02 | 3.62E+02 | 4.36E+02 | 5.27E+02 | 4.42E+02 | 1.23E+03 | 8.17E+02 | 1.05E+03 | 4.56E+02 | 1.05E+03 | 1.22E+03 | 1.28E+03 | 8.61E+02 | 2.60E+03 | 7.19E+02 | 2.02026378 | 0.003604510447746673 | 2.7008 | 1.4334 | up |
| Lipid-Q-P-0061 | C47H76O2 | CE(20:4) | ST | CE | LMST01020014 | 3.47E+02 | 1.66E+02 | 5.44E+01 | 2.29E+03 | 2.70E+02 | 2.38E+02 | 2.53E+02 | 2.33E+02 | 1.98E+02 | 3.45E+02 | 2.70E+04 | 3.92E+04 | 2.86E+04 | 3.64E+02 | 3.32E+04 | 3.54E+04 | 3.39E+04 | 1.29E+04 | 2.30E+04 | 2.39E+02 | 2.01775549 | 6.371298353783264e-4 | 53.2342 | 5.7343 | up |
| Lipid-Q-P-0891 | C44H78O6 | TG(41:3)_18:2 | GL | TG | - | 9.60E-01 | 4.81E-01 | 1.93E+00 | N/A | 1.13E-01 | 2.48E-01 | 3.07E-01 | 1.96E-01 | 8.42E-02 | N/A | N/A | N/A | N/A | N/A | N/A | N/A | 3.08E-02 | N/A | N/A | N/A | 2.01102683 | N/A | 0.0571 | -4.1304 | down |
| Lipid-Q-P-0046 | C47H82O2 | CE(20:1) | ST | CE | LMST01020011 | 3.77E+00 | 4.45E+00 | 2.71E+01 | 9.91E+02 | 3.29E+00 | 6.10E+00 | 6.49E+00 | 1.24E+01 | 3.28E+00 | 1.24E+01 | 8.09E+03 | 7.37E+03 | 6.14E+03 | 1.69E+01 | 2.00E+04 | 2.13E+04 | 2.89E+04 | 1.02E+04 | 2.06E+03 | 3.78E+01 | 2.00383605 | 0.009043829388296768 | 97.4351 | 6.6064 | up |
| Lipid-Q-P-0051 | C45H76O2 | CE(18:2) | ST | CE | LMST01020008 | 8.95E+02 | 3.11E+02 | 1.69E+02 | 4.28E+03 | 1.30E+03 | 1.09E+03 | 8.99E+02 | 9.79E+02 | 4.42E+02 | 1.29E+03 | 6.58E+04 | 5.78E+04 | 5.47E+04 | 1.13E+03 | 7.04E+04 | 7.82E+04 | 1.06E+05 | 7.64E+04 | 8.70E+03 | 1.34E+03 | 2.00351709 | 0.0016079211025999387 | 44.6404 | 5.4803 | up |
| Lipid-Q-P-0718 | C41H78O6 | TG(38:0)_12:0 | GL | TG | - | 8.54E-01 | 7.78E-01 | 2.04E+00 | 2.89E-01 | 6.33E-01 | 5.88E-01 | 6.69E-01 | 5.20E-01 | 4.28E-01 | 1.24E+00 | 2.91E-01 | 2.05E-01 | 1.84E-01 | 4.03E-01 | 2.27E-01 | 2.23E-01 | 2.61E-01 | 2.70E-01 | 1.42E-01 | 2.08E-01 | 1.99632564 | 0.006508393023481188 | 0.3004 | -1.735 | down |
| Lipid-Q-P-0047 | C49H86O2 | CE(22:1) | ST | CE | LMST01020025 | 1.16E+00 | 1.79E+00 | 1.03E+01 | 4.65E+02 | 9.55E-01 | 1.67E+00 | 2.21E+00 | 3.98E+00 | 1.17E+00 | 2.92E+00 | 4.61E+03 | 3.81E+03 | 3.80E+03 | 5.70E+00 | 1.26E+04 | 1.18E+04 | 1.34E+04 | 5.29E+03 | 7.89E+02 | 1.08E+01 | 1.98371897 | 0.007889931600162284 | 114.0235 | 6.8332 | up |
| Lipid-Q-P-0025 | C23H41NO4 | CAR16:2 | FA | CAR | LMFA07070021 | 7.75E-02 | 6.33E-02 | 1.65E-02 | 3.66E+00 | 5.88E-02 | 5.09E-02 | 3.98E-02 | 5.17E-01 | 5.17E-01 | 1.72E+00 | 1.21E+00 | 1.99E+00 | 1.45E+00 | 8.77E-01 | 1.70E+00 | 1.60E+00 | 3.00E+00 | 7.02E-01 | 4.78E+00 | 7.43E-01 | 1.98035872 | 0.051274901479925646 | 2.6833 | 1.424 | up |
| Lipid-Q-P-0725 | C46H88O6 | TG(43:0)_16:0 | GL | TG | LMGL03013268 | 3.58E+00 | 2.94E+00 | 6.74E+00 | 2.32E+00 | 3.16E+00 | 3.30E+00 | 4.11E+00 | 5.58E+00 | 3.12E+00 | 5.11E+00 | 2.07E+00 | 1.72E+00 | 1.97E+00 | 2.32E+00 | 1.52E+00 | 1.11E+00 | 2.20E+00 | 1.86E+00 | 1.16E+00 | 2.35E+00 | 1.97371669 | 6.820691394047062e-4 | 0.4577 | -1.1275 | down |
| Lipid-Q-P-0043 | C44H76O2 | CE(17:1) | ST | CE | LMST01020023 | N/A | N/A | N/A | 3.12E+01 | N/A | N/A | N/A | N/A | 1.64E+00 | N/A | 5.18E+02 | 5.03E+02 | 4.43E+02 | N/A | 5.61E+02 | 5.13E+02 | 8.79E+02 | 4.09E+02 | 2.08E+02 | 2.38E+00 | 1.97311375 | N/A | 27.3232 | 4.7721 | up |
| Lipid-Q-P-0017 | C21H39NO5 | CAR14:1-OH | FA | CAR | - | 2.52E-02 | 2.70E-02 | 9.37E-03 | 2.99E-01 | 1.65E-02 | 1.44E-02 | 9.13E-03 | 6.34E-02 | 6.56E-02 | 2.01E-01 | 1.24E+00 | 5.53E-01 | 1.00E+00 | 1.02E-01 | 1.23E-01 | 1.14E-01 | 1.98E-01 | 5.14E-01 | 6.56E-01 | 1.99E-01 | 1.96945735 | 0.01231862337876403 | 6.4269 | 2.6841 | up |
| Lipid-Q-P-0465 | C52H94NO8P | PC(44:5) | GP | PC | - | 7.16E-02 | 9.18E-02 | 4.95E-02 | 2.27E-01 | 1.01E-01 | 9.65E-02 | 8.31E-02 | 8.72E-02 | 9.39E-02 | 1.15E-01 | 1.74E-01 | 2.03E-01 | 1.97E-01 | 1.13E-01 | 3.21E-01 | 2.65E-01 | 2.73E-01 | 1.62E-01 | 6.01E-01 | 1.35E-01 | 1.96462094 | 0.01132111092725696 | 2.4043 | 1.2656 | up |
| Lipid-Q-P-0419 | C39H78NO8P | PC(31:0) | GP | PC | LMGP01010532 | 1.02E+01 | 1.36E+01 | 6.60E+00 | 4.05E+01 | 1.37E+01 | 1.28E+01 | 1.50E+01 | 1.61E+01 | 2.29E+01 | 2.15E+01 | 3.47E+01 | 3.56E+01 | 5.70E+01 | 2.69E+01 | 3.38E+01 | 2.87E+01 | 2.95E+01 | 2.29E+01 | 5.70E+01 | 2.24E+01 | 1.9616367 | 0.0025563225071569312 | 2.0168 | 1.0121 | up |
| Lipid-Q-P-0771 | C46H86O6 | TG(43:1)_16:1 | GL | TG | LMGL03013269 | 2.64E+00 | 2.27E+00 | 6.12E+00 | 4.61E-01 | 1.29E+00 | 1.33E+00 | 1.44E+00 | 1.50E+00 | 1.10E+00 | 2.47E+00 | 6.92E-01 | 6.01E-01 | 4.96E-01 | 7.83E-01 | 5.22E-01 | 2.23E-01 | 3.75E-01 | 3.52E-01 | 3.51E-01 | 1.65E+00 | 1.95478535 | 0.017446805329165916 | 0.293 | -1.771 | down |
| Lipid-Q-P-0005 | C21H41NO4 | CAR14:0 | FA | CAR | LMFA07070102 | 1.71E-01 | 2.44E-01 | 3.86E-02 | 9.02E+00 | 3.09E-01 | 2.17E-01 | 1.80E-01 | 1.12E+00 | 1.37E+00 | 4.34E+00 | 2.85E+00 | 4.26E+00 | 3.80E+00 | 2.68E+00 | 2.34E+00 | 3.93E+00 | 3.71E+00 | 1.63E+00 | 7.53E+00 | 2.59E+00 | 1.95266712 | 0.10188427912270744 | 2.0758 | 1.0537 | up |
| Lipid-Q-N-0084 | C24H46O2 | FFA(24:1) | FA | FFA | LMFA01030092 | 1.42E+00 | 3.63E+00 | 3.00E+00 | 1.34E+01 | 5.09E+00 | 8.16E+00 | 6.63E+00 | 6.64E+00 | 4.46E+00 | 5.18E+00 | 2.01E+01 | 1.37E+01 | 1.71E+01 | 6.16E+00 | 5.80E+01 | 3.99E+01 | 3.35E+01 | 4.08E+01 | 4.45E+01 | 7.82E+00 | 1.95024455 | 0.0028793743537159928 | 4.8837 | 2.288 | up |
| Lipid-Q-P-0027 | C27H49NO4 | CAR 20:2 | FA | CAR | - | 1.68E-01 | 1.90E-01 | 4.13E-02 | 3.98E+00 | 5.58E-01 | 4.18E-01 | 4.07E-01 | 1.22E+00 | 1.58E+00 | 4.89E+00 | 2.21E+00 | 8.52E+00 | 1.95E+00 | 2.92E+00 | 2.93E+00 | 5.23E+00 | 3.57E+00 | 2.24E+00 | 4.41E+00 | 2.95E+00 | 1.93718354 | 0.011103884057864064 | 2.7449 | 1.4568 | up |
| Lipid-Q-N-0083 | C22H42O2 | FFA(22:1) | FA | FFA | LMFA01030088 | 4.27E+00 | 7.86E+00 | 6.15E+00 | 2.39E+01 | 5.93E+00 | 8.83E+00 | 1.17E+01 | 5.38E+00 | 6.80E+00 | 8.60E+00 | 2.87E+01 | 3.05E+01 | 3.22E+01 | 7.44E+00 | 1.20E+02 | 7.03E+01 | 1.01E+02 | 5.34E+01 | 4.14E+01 | 1.28E+01 | 1.93501574 | 0.006829827656929345 | 5.5671 | 2.4769 | up |
| Lipid-Q-P-0033 | C43H76O2 | CE(16:0) | ST | CE | LMST01020005 | 9.94E+01 | 3.65E+01 | 4.79E+01 | 5.55E+02 | 3.83E+01 | 3.18E+01 | 3.46E+01 | 5.22E+01 | 2.90E+01 | 7.44E+01 | 3.26E+03 | 2.70E+03 | 3.07E+03 | 5.95E+01 | 4.06E+03 | 2.70E+03 | 3.75E+03 | 2.10E+03 | 9.41E+02 | 4.65E+01 | 1.92974996 | 0.0010603200583290979 | 22.7122 | 4.5054 | up |
| Lipid-Q-P-0045 | C46H80O2 | CE(19:1) | ST | CE | - | N/A | N/A | N/A | 2.32E+01 | N/A | N/A | N/A | N/A | N/A | N/A | 4.84E+02 | 4.50E+02 | 3.58E+02 | N/A | 5.94E+02 | 7.37E+02 | 8.68E+02 | 3.88E+02 | 1.66E+02 | N/A | 1.92000386 | N/A | 21.8174 | 4.4474 | up |
| Lipid-Q-P-0052 | C46H78O2 | CE(19:2) | ST | CE | - | N/A | N/A | N/A | 1.23E+01 | N/A | N/A | N/A | N/A | N/A | N/A | 1.67E+02 | 1.65E+02 | 1.23E+02 | N/A | 2.57E+02 | 2.91E+02 | 3.35E+02 | 2.04E+02 | 5.34E+01 | N/A | 1.91775081 | N/A | 16.1664 | 4.0149 | up |
| Lipid-Q-P-0720 | C43H82O6 | TG(40:0)_14:0 | GL | TG | - | 4.89E+00 | 7.70E+00 | 1.68E+01 | 7.24E-01 | 1.47E+00 | 1.39E+00 | 1.19E+00 | 1.20E+00 | 1.98E+00 | 3.09E+00 | 4.49E-01 | 2.84E-01 | 3.98E-01 | 7.17E-01 | 7.68E-01 | 2.90E-01 | 9.60E-01 | 2.12E-01 | 5.28E-01 | 7.71E-01 | 1.91625473 | 0.05288441464349818 | 0.1331 | -2.9094 | down |
| Lipid-Q-P-0019 | C23H43NO5 | CAR16:1-OH | FA | CAR | LMFA07070044 | 1.92E-02 | 4.29E-02 | N/A | 7.83E-01 | 4.04E-02 | 3.28E-02 | 1.76E-02 | 8.87E-02 | 2.35E-01 | 3.59E-01 | 1.72E+00 | 9.81E-01 | 1.56E+00 | 2.18E-01 | 4.33E-01 | 4.16E-01 | 6.01E-01 | 1.44E+00 | 2.48E+00 | 5.20E-01 | 1.91386497 | 0.005121912685060643 | 5.7625 | 2.5267 | up |
| Lipid-Q-P-0427 | C40H78NO8P | PC(32:1) | GP | PC | LMGP01010566 | 6.71E+01 | 6.73E+01 | 3.83E+01 | 2.37E+02 | 1.27E+02 | 1.34E+02 | 1.37E+02 | 1.27E+02 | 1.74E+02 | 1.50E+02 | 2.97E+02 | 2.34E+02 | 3.21E+02 | 9.90E+01 | 3.53E+02 | 2.94E+02 | 3.18E+02 | 2.35E+02 | 3.22E+02 | 1.67E+02 | 1.91316069 | 4.120556029907072e-4 | 2.0949 | 1.0669 | up |
| Lipid-Q-P-0034 | C44H78O2 | CE(17:0) | ST | CE | LMST01020026 | N/A | N/A | N/A | 1.18E+01 | N/A | N/A | N/A | N/A | N/A | N/A | 7.59E+01 | 5.23E+01 | 5.61E+01 | N/A | 1.15E+02 | 5.18E+01 | 7.04E+01 | 4.75E+01 | 2.58E+01 | N/A | 1.90206465 | N/A | 5.2473 | 2.3916 | up |
| Lipid-Q-P-0002 | C19H37NO4 | CAR12:0 | FA | CAR | LMFA07070062 | 8.90E-02 | 3.63E-02 | 1.44E-02 | 9.49E-01 | 2.05E-02 | 1.83E-02 | 1.63E-02 | 2.06E-01 | 1.36E-01 | 8.09E-01 | 3.93E-01 | 6.52E-01 | 5.08E-01 | 5.49E-01 | 3.11E-01 | 5.08E-01 | 4.95E-01 | 1.92E-01 | 8.55E-01 | 3.12E-01 | 1.88137469 | 0.06883945712492638 | 2.0808 | 1.0571 | up |
| Lipid-Q-P-0015 | C19H35NO5 | CAR12:1-OH | FA | CAR | LMFA07070024 | 9.79E-03 | 9.13E-03 | 5.29E-03 | 5.08E-02 | 8.33E-03 | 6.87E-03 | 5.57E-03 | 2.07E-02 | 1.54E-02 | 5.48E-02 | 2.43E-01 | 1.09E-01 | 1.94E-01 | 2.10E-02 | 2.49E-02 | 2.80E-02 | 3.85E-02 | 9.86E-02 | 7.99E-02 | 4.33E-02 | 1.87431268 | 0.018928289774381397 | 4.7093 | 2.2355 | up |
| Lipid-Q-P-0020 | C25H47NO4 | CAR18:1 | FA | CAR | LMFA07070096 | 4.17E+00 | 2.71E+00 | 5.74E-01 | 2.56E+01 | 4.39E+00 | 2.78E+00 | 2.08E+00 | 8.01E+00 | 2.00E+01 | 2.94E+01 | 1.81E+01 | 4.13E+01 | 1.89E+01 | 1.74E+01 | 2.63E+01 | 2.81E+01 | 3.53E+01 | 8.27E+00 | 2.73E+01 | 1.65E+01 | 1.87209588 | 0.00798595444432283 | 2.3801 | 1.251 | up |
| Lipid-Q-P-0026 | C25H45NO4 | CAR18:2 | FA | CAR | LMFA07070092 | 2.34E+00 | 1.09E+00 | 3.22E-01 | 1.77E+01 | 2.61E+00 | 1.95E+00 | 1.48E+00 | 6.39E+00 | 1.04E+01 | 1.63E+01 | 1.43E+01 | 1.94E+01 | 1.44E+01 | 8.72E+00 | 1.93E+01 | 2.22E+01 | 3.00E+01 | 4.80E+00 | 1.37E+01 | 6.41E+00 | 1.87157245 | 0.009624239205266475 | 2.5264 | 1.3371 | up |
| Lipid-Q-P-0513 | C50H90NO7P | PC(O-42:6) | GP | PC-O | LMGP01020244 | 7.16E-01 | 7.76E-01 | 1.01E-01 | 1.42E+00 | 4.01E-01 | 2.76E-01 | 3.52E-01 | 6.19E-01 | 4.19E-01 | 9.10E-01 | 8.69E-01 | 1.02E+00 | 8.15E-01 | 6.11E-01 | 1.49E+00 | 1.79E+00 | 1.80E+00 | 1.15E+00 | 3.71E+00 | 1.46E+00 | 1.85986513 | 0.014128446990748913 | 2.457 | 1.2969 | up |
| Lipid-Q-P-0477 | C38H78NO7P | PC(O-30:0) | GP | PC-O | LMGP01020178 | 1.88E-01 | 1.69E-01 | 1.25E-01 | 2.89E-01 | 1.62E-01 | 1.69E-01 | 1.44E-01 | 1.34E-01 | 1.49E-01 | 1.44E-01 | 1.20E+00 | 6.08E-01 | 1.54E+00 | 1.75E-01 | 1.96E+00 | 1.17E+00 | 2.14E+00 | 1.73E+00 | 1.53E+00 | 1.07E-01 | 1.85365432 | 0.0011778561026968212 | 7.2577 | 2.8595 | up |
| Lipid-Q-P-0018 | C23H43NO4 | CAR16:1 | FA | CAR | LMFA07070097 | 4.73E-01 | 4.21E-01 | 7.46E-02 | 1.16E+01 | 4.82E-01 | 2.84E-01 | 2.30E-01 | 1.49E+00 | 5.22E+00 | 7.59E+00 | 3.86E+00 | 8.57E+00 | 4.38E+00 | 3.90E+00 | 4.94E+00 | 5.58E+00 | 7.81E+00 | 1.74E+00 | 1.27E+01 | 3.58E+00 | 1.84765447 | 0.08819468472604013 | 2.0492 | 1.0351 | up |
| Lipid-Q-P-0012 | C25H49NO5 | CAR18-OH | FA | CAR | LMFA07070043 | 1.82E-02 | 5.77E-02 | 1.02E-02 | 9.28E-01 | 3.26E-01 | 2.76E-01 | 2.39E-01 | 3.53E-01 | 6.90E-01 | 8.50E-01 | 1.52E+00 | 1.35E+00 | 1.30E+00 | 6.57E-01 | 6.37E-01 | 9.62E-01 | 8.58E-01 | 1.90E+00 | 2.15E+00 | 7.77E-01 | 1.84520506 | 6.920510021840868e-4 | 3.234 | 1.6933 | up |
| Lipid-Q-P-0730 | C48H92O6 | TG(45:0)_16:0 | GL | TG | LMGL03014167 | 1.88E+01 | 1.84E+01 | 4.13E+01 | 8.12E+00 | 1.63E+01 | 1.39E+01 | 1.48E+01 | 1.22E+01 | 9.27E+00 | 2.20E+01 | 1.05E+01 | 7.95E+00 | 8.82E+00 | 1.31E+01 | 7.20E+00 | 4.95E+00 | 6.35E+00 | 6.20E+00 | 7.73E+00 | 1.40E+01 | 1.83255844 | 0.016539761871887312 | 0.4962 | -1.011 | down |
| Lipid-Q-N-0001 | C26H45NO8S2 | TLCA-3S | ST | BA | - | 1.26E+01 | 1.66E+01 | 2.49E+01 | 4.46E+00 | 5.14E+00 | 7.06E+00 | 3.76E+00 | 5.93E+00 | 2.56E+00 | 2.46E+00 | 1.73E+00 | 4.54E+00 | 2.96E+00 | 2.73E+00 | 2.99E+00 | 1.76E+00 | 1.88E+00 | 3.15E+00 | 1.03E+00 | 1.44E+00 | 1.81019519 | 0.026945309680336323 | 0.2832 | -1.8201 | down |
| Lipid-Q-P-0358 | C25H52NO7P | LPE(20:0/0:0) | GP | LPE | LMGP02050045 | 2.94E+00 | 1.76E+00 | 2.46E+00 | 6.33E-01 | 8.77E-01 | 1.12E+00 | 1.20E+00 | 7.15E-01 | 5.17E-01 | 4.75E-01 | 2.53E-01 | 4.84E-01 | 3.62E-01 | 3.67E-01 | 6.28E-01 | 5.53E-01 | 6.27E-01 | 5.00E-01 | 3.33E-01 | 2.38E-01 | 1.80979358 | 0.012867384803655067 | 0.3425 | -1.5458 | down |
| Lipid-Q-N-0123 | C24H46NO9P | LPS(18:1) | GP | LPS | LMGP03050001 | 1.13E+02 | 1.36E+02 | 4.75E+01 | 9.13E+01 | 3.60E+01 | 6.19E+01 | 5.14E+01 | 4.95E+01 | 6.85E+01 | 7.49E+01 | 2.24E+01 | 2.51E+01 | 3.61E+01 | 2.84E+01 | 1.40E+01 | 7.62E+00 | 1.25E+01 | 2.80E+01 | 1.77E+01 | 2.64E+01 | 1.80866311 | 5.572712781592213e-4 | 0.299 | -1.7418 | down |
| Lipid-Q-P-0185 | C45H86O5 | DG(18:1_24:0) | GL | DG | - | N/A | N/A | N/A | 3.45E-01 | 5.28E-02 | N/A | N/A | N/A | N/A | N/A | 1.59E+00 | 1.28E+00 | 2.36E+00 | N/A | 7.24E+00 | 7.22E+00 | 6.84E+00 | 2.51E+00 | 1.52E+00 | N/A | 1.80017195 | N/A | 19.1925 | 4.2625 | up |
| Lipid-Q-N-0093 | C22H36O2 | FFA(22:4) | FA | FFA | LMFA01030177 | 3.78E+00 | 1.41E+01 | 1.38E+01 | 9.80E+01 | 2.45E+01 | 3.38E+01 | 2.91E+01 | 1.41E+01 | 1.96E+01 | 1.61E+01 | 8.64E+01 | 8.67E+01 | 9.97E+01 | 1.47E+01 | 1.12E+02 | 9.47E+01 | 9.20E+01 | 7.26E+01 | 1.69E+02 | 3.08E+01 | 1.79837804 | 0.0019477829399283922 | 3.2131 | 1.684 | up |
| Lipid-Q-P-0561 | C47H84NO8P | PE(42:5) | GP | PE | - | 5.32E-01 | 1.57E+00 | 3.77E-01 | 3.22E+00 | 2.13E+00 | 2.35E+00 | 2.27E+00 | 1.97E+00 | 2.00E+00 | 2.37E+00 | 3.36E+00 | 2.88E+00 | 3.15E+00 | 1.41E+00 | 1.41E+01 | 7.97E+00 | 1.31E+01 | 8.56E+00 | 6.31E+00 | 2.92E+00 | 1.78915337 | 0.011333717561458125 | 3.3937 | 1.7629 | up |
| Lipid-Q-P-0604 | C43H76NO7P | PE(P-38:5) | GP | PE-P | LMGP02030056 | 1.75E+02 | 2.60E+02 | 6.85E+01 | 6.79E+02 | 3.90E+02 | 4.25E+02 | 3.60E+02 | 4.10E+02 | 5.80E+02 | 5.73E+02 | 6.53E+02 | 5.38E+02 | 5.37E+02 | 3.60E+02 | 1.12E+03 | 8.72E+02 | 1.20E+03 | 7.40E+02 | 1.36E+03 | 5.20E+02 | 1.78765477 | 0.005602980701786574 | 2.017 | 1.0122 | up |
| Lipid-Q-P-0016 | C21H39NO4 | CAR14:1 | FA | CAR | LMFA07070057 | 2.78E-01 | 1.55E-01 | 4.58E-02 | 6.12E+00 | 4.00E-02 | 3.50E-02 | 3.28E-02 | 3.84E-01 | 4.05E-01 | 1.70E+00 | 2.41E+00 | 2.48E+00 | 3.07E+00 | 1.51E+00 | 6.47E-01 | 5.68E-01 | 1.23E+00 | 7.74E-01 | 7.48E+00 | 1.07E+00 | 1.7855176 | 0.19186406792162103 | 2.3079 | 1.2066 | up |
| Lipid-Q-P-0042 | C43H74O2 | CE(16:1) | ST | CE | LMST01020006 | 9.54E+00 | N/A | N/A | 1.96E+02 | 1.77E+01 | 1.19E+01 | 1.25E+01 | 1.56E+01 | 1.25E+01 | 2.42E+01 | 2.08E+03 | 1.96E+03 | 1.64E+03 | 2.36E+01 | 2.86E+03 | 3.45E+03 | 4.81E+03 | 2.04E+03 | 1.07E+03 | 2.14E+01 | 1.78434281 | 0.002295968479896078 | 53.2364 | 5.7343 | up |
| Lipid-Q-P-0426 | C39H76NO8P | PC(31:1) | GP | PC | LMGP01010535 | 1.92E-01 | 2.47E-01 | 1.52E-01 | 1.32E+00 | 3.63E-01 | 6.10E-01 | 6.25E-01 | 3.25E-01 | 7.71E-01 | 5.63E-01 | 1.23E+00 | 1.10E+00 | 1.33E+00 | 2.84E-01 | 1.70E+00 | 1.40E+00 | 1.51E+00 | 7.79E-01 | 2.90E+00 | 5.68E-01 | 1.77684034 | 0.009883077885588619 | 2.4743 | 1.307 | up |
| Lipid-Q-P-0357 | C23H48NO7P | LPE(18:0/0:0) | GP | LPE | LMGP02050001 | 1.74E+01 | 1.08E+01 | 9.22E+00 | 6.02E+01 | 6.90E+01 | 9.94E+01 | 1.11E+02 | 4.87E+01 | 2.73E+01 | 2.06E+01 | 1.38E+00 | 4.39E+00 | 1.96E+00 | 4.91E+00 | 6.13E+00 | 4.02E+00 | 4.94E+00 | 3.15E+00 | 3.23E+00 | 5.68E+00 | 1.76814557 | 0.00472181567817237 | 0.084 | -3.5735 | down |
| Lipid-Q-P-0562 | C49H88NO8P | PE(44:5) | GP | PE | - | 1.42E-01 | 2.97E-01 | 1.47E-01 | 8.97E-01 | 5.14E-01 | 5.71E-01 | 6.01E-01 | 5.12E-01 | 4.59E-01 | 5.98E-01 | 7.79E-01 | 7.02E-01 | 6.81E-01 | 4.45E-01 | 5.03E+00 | 2.38E+00 | 4.12E+00 | 1.59E+00 | 3.04E+00 | 6.12E-01 | 1.76592676 | 0.020330304237473 | 4.0875 | 2.0312 | up |
| Lipid-Q-P-0024 | C21H37NO4 | CAR14:2 | FA | CAR | LMFA07070020 | 1.84E-01 | 7.35E-02 | 2.17E-02 | 1.85E+00 | 9.84E-03 | 1.23E-02 | 1.00E-02 | 2.09E-01 | 5.08E-02 | 4.82E-01 | 1.28E+00 | 7.92E-01 | 1.47E+00 | 4.79E-01 | 2.80E-01 | 1.95E-01 | 6.27E-01 | 2.49E-01 | 2.14E+00 | 3.58E-01 | 1.76247745 | 0.08461608937558193 | 2.7093 | 1.4379 | up |
| Lipid-Q-P-0580 | C37H72NO7P | PE(P-32:1) | GP | PE-P | - | 3.00E+00 | 3.33E+00 | 2.81E+00 | 3.56E+00 | 2.66E+00 | 2.48E+00 | 2.49E+00 | 2.94E+00 | 3.14E+00 | 3.25E+00 | 7.79E+00 | 4.17E+00 | 7.86E+00 | 2.27E+00 | 1.06E+01 | 9.22E+00 | 8.32E+00 | 1.31E+01 | 4.58E+00 | 3.62E+00 | 1.75564485 | 0.0038096200738809392 | 2.414 | 1.2714 | up |
| Lipid-Q-P-0476 | C50H84NO8P | PC(42:8) | GP | PC | LMGP01011865 | 4.38E-01 | 6.84E-01 | 9.32E-02 | 1.78E+00 | 5.97E-01 | 5.90E-01 | 6.78E-01 | 5.12E-01 | 5.41E-01 | 1.11E+00 | 9.40E-01 | 1.61E+00 | 1.26E+00 | 1.00E+00 | 9.58E-01 | 1.26E+00 | 9.46E-01 | 1.02E+00 | 4.19E+00 | 1.41E+00 | 1.73348065 | 0.04693386951169717 | 2.0764 | 1.0541 | up |
| Lipid-Q-P-0178 | C39H74O5 | DG(16:0_20:1) | GL | DG | LMGL02010048 | 2.62E-01 | 1.89E+00 | 2.05E+00 | 1.35E+01 | 7.82E-01 | 2.16E+00 | 2.61E+00 | 8.81E-01 | 1.51E+00 | 4.36E-01 | 3.04E+01 | 1.92E+01 | 1.42E+01 | 1.03E+00 | 2.87E+01 | 1.24E+01 | 1.46E+01 | 2.22E+01 | 1.05E+01 | 2.83E+00 | 1.72753922 | 0.002172902768954192 | 5.9811 | 2.5804 | up |
| Lipid-Q-N-0090 | C22H38O2 | FFA(22:3) | FA | FFA | LMFA01030161 | 1.24E+00 | 4.24E+00 | 3.73E+00 | 4.08E+01 | 7.15E+00 | 9.12E+00 | 9.18E+00 | 3.02E+00 | 4.98E+00 | 6.64E+00 | 1.30E+01 | 1.69E+01 | 1.76E+01 | 5.85E+00 | 3.20E+01 | 2.23E+01 | 2.65E+01 | 1.75E+01 | 5.75E+01 | 6.85E+00 | 1.69216484 | 0.050191089004709005 | 2.3955 | 1.2603 | up |
| Lipid-Q-N-0008 | C24H38O3 | 6,7-DKLCA | ST | BA | - | 3.50E+01 | 7.19E+01 | 1.03E+02 | 1.13E+02 | 4.53E+01 | 9.17E+01 | 5.38E+01 | 5.88E+01 | 4.98E+01 | 4.13E+01 | 2.87E+01 | 2.28E+01 | 3.78E+01 | 5.08E+01 | 3.03E+01 | 2.42E+01 | 2.34E+01 | 3.75E+01 | 2.17E+01 | 3.50E+01 | 1.68416574 | 0.0026690534995415754 | 0.4704 | -1.088 | down |
| Lipid-Q-P-0067 | C49H78O2 | CE(22:5) | ST | CE | LMST01020031 | 2.93E+00 | N/A | 6.55E+00 | 1.54E+03 | 3.48E+00 | 3.56E+00 | 5.53E+00 | N/A | N/A | 1.48E+01 | 9.95E+02 | 2.26E+03 | 2.14E+03 | 3.11E+01 | 7.14E+03 | 8.42E+03 | 1.71E+04 | 1.40E+03 | 1.32E+03 | 3.07E+01 | 1.68103063 | 0.05063728902377995 | 18.1337 | 4.1806 | up |
| Lipid-Q-P-0962 | C47H82O6 | TG(44:4)_18:2 | GL | TG | - | 9.27E+00 | 7.97E+00 | 2.98E+01 | 5.31E-01 | 1.47E+00 | 1.10E+00 | 1.14E+00 | 6.21E-01 | 1.65E-01 | 7.01E-01 | 6.27E-02 | 7.72E-02 | 1.05E-01 | 3.28E-02 | 2.21E+00 | 3.33E-02 | 6.78E-01 | 3.53E-02 | N/A | 9.19E-03 | 1.64865581 | 0.1265550353586615 | 0.0684 | -3.8699 | down |
| Lipid-Q-P-0769 | C45H84O6 | TG(42:1)_14:0 | GL | TG | LMGL03013212 | 1.51E+01 | 1.59E+01 | 5.49E+01 | 2.83E-01 | 9.42E-01 | 1.27E+00 | 1.46E+00 | 1.03E+00 | 7.67E-01 | 1.86E+00 | 4.85E-01 | 4.02E-01 | 3.25E-01 | 4.66E-01 | 2.04E+00 | 2.74E-01 | 1.15E+00 | 3.46E-01 | 3.59E-01 | 6.10E-01 | 1.64562141 | 0.14190715794103217 | 0.0691 | -3.8552 | down |
| Lipid-Q-N-0086 | C20H36O2 | FFA(20:2) | FA | FFA | LMFA01030130 | 6.66E+00 | 1.66E+01 | 2.72E+01 | 1.21E+02 | 4.05E+01 | 6.00E+01 | 5.86E+01 | 1.80E+01 | 2.16E+01 | 1.90E+01 | 1.38E+02 | 1.15E+02 | 1.48E+02 | 1.58E+01 | 2.04E+02 | 1.42E+02 | 1.63E+02 | 1.80E+02 | 1.33E+02 | 3.56E+01 | 1.64530567 | 0.0010684691248607275 | 3.27 | 1.7093 | up |
| Lipid-Q-P-0500 | C44H82NO7P | PC(O-36:4) | GP | PC-O | LMGP01020054 | 3.93E+00 | 4.71E+00 | 1.47E+00 | 3.66E+01 | 1.14E+01 | 1.13E+01 | 1.23E+01 | 9.89E+00 | 1.54E+01 | 9.65E+00 | 1.74E+01 | 1.26E+01 | 1.46E+01 | 8.16E+00 | 4.94E+01 | 3.87E+01 | 5.70E+01 | 1.92E+01 | 9.26E+01 | 9.08E+00 | 1.64166611 | 0.050286430039024874 | 2.7317 | 1.4498 | up |
| Lipid-Q-P-0538 | C45H86NO8P | PE(40:2) | GP | PE | LMGP02011082 | 7.36E-01 | 2.42E+00 | 5.79E-01 | 1.30E+00 | 7.05E-01 | 7.30E-01 | 7.11E-01 | 9.28E-01 | 9.10E-01 | 2.23E+00 | 1.99E+00 | 2.73E+00 | 2.63E+00 | 9.75E-01 | 4.89E+00 | 4.83E+00 | 4.74E+00 | 2.10E+00 | 8.96E-01 | 1.08E+00 | 1.63360926 | 0.01489355468954955 | 2.3878 | 1.2557 | up |
| Lipid-Q-P-0021 | C27H51NO4 | CAR 20:1 | FA | CAR | - | 1.15E-02 | N/A | N/A | 1.16E-01 | 2.39E-02 | 1.58E-02 | 1.63E-02 | 4.41E-02 | 5.36E-02 | 1.65E-01 | 7.29E-02 | 2.89E-01 | 6.57E-02 | 9.24E-02 | 9.92E-02 | 1.71E-01 | 1.22E-01 | 7.93E-02 | 1.52E-01 | 9.37E-02 | 1.63265877 | 0.032729751051153486 | 2.2168 | 1.1485 | up |
| Lipid-Q-P-0756 | C59H114O6 | TG(56:0)_24:0 | GL | TG | - | 3.83E+00 | 9.43E-01 | 2.49E+00 | 1.41E+00 | 4.00E-01 | 5.01E-01 | 3.79E-01 | 4.56E-01 | 3.88E-01 | 8.34E-01 | 3.48E+01 | 1.22E+01 | 1.77E+01 | 5.88E-01 | 1.57E+01 | 1.78E+01 | 2.07E+01 | 2.55E+01 | 2.76E+00 | 8.45E-01 | 1.63201579 | 0.003661066881914616 | 12.763 | 3.6739 | up |
| Lipid-Q-P-0761 | C63H122O6 | TG(60:0)_20:0 | GL | TG | LMGL03010003 | 7.65E-04 | N/A | N/A | N/A | N/A | N/A | N/A | N/A | N/A | N/A | 2.38E-02 | N/A | 2.48E-03 | N/A | 4.94E-02 | 3.43E-02 | 5.34E-02 | 9.62E-03 | 1.82E-02 | N/A | 1.62771098 | N/A | 35.6788 | 5.157 | up |
| Lipid-Q-P-0759 | C61H118O6 | TG(58:0)_18:0 | GL | TG | - | 1.10E+00 | 8.71E-02 | 3.79E-01 | 3.98E-01 | 1.75E-01 | 1.24E-01 | 2.06E-01 | 1.84E-01 | 8.81E-02 | 2.43E-01 | 4.76E+00 | 1.33E+00 | 2.24E+00 | 1.00E-01 | 4.07E+00 | 5.08E+00 | 6.64E+00 | 2.62E+00 | 9.05E-01 | 2.14E-01 | 1.62469781 | 0.00655304861587684 | 9.3657 | 3.2274 | up |
| Lipid-Q-P-0213 | C43H78O5 | DG(18:1_22:2) | GL | DG | - | N/A | N/A | N/A | 3.07E-01 | N/A | N/A | N/A | N/A | N/A | N/A | 2.46E+00 | 1.83E+00 | 2.12E+00 | N/A | 1.62E+00 | 8.41E-02 | 4.47E-01 | 2.92E+00 | N/A | N/A | 1.62093659 | N/A | 5.3447 | 2.4181 | up |
| Lipid-Q-N-0082 | C20H38O2 | FFA(20:1) | FA | FFA | LMFA01030082 | 8.90E+00 | 1.92E+01 | 3.86E+01 | 8.82E+01 | 2.26E+01 | 3.76E+01 | 4.47E+01 | 1.23E+01 | 3.03E+01 | 1.13E+01 | 8.29E+01 | 7.77E+01 | 1.05E+02 | 1.43E+01 | 3.49E+02 | 2.49E+02 | 2.84E+02 | 1.66E+02 | 1.10E+02 | 2.79E+01 | 1.60640657 | 0.010291313286888822 | 4.6778 | 2.2258 | up |
| Lipid-Q-P-0505 | C44H80NO7P | PC(O-36:5) | GP | PC-O | LMGP01020259 | 4.50E+01 | 5.87E+01 | 2.06E+01 | 3.38E+02 | 1.11E+02 | 1.21E+02 | 1.30E+02 | 1.05E+02 | 1.49E+02 | 9.55E+01 | 1.49E+02 | 1.25E+02 | 1.43E+02 | 8.77E+01 | 4.98E+02 | 3.98E+02 | 6.12E+02 | 1.88E+02 | 1.10E+03 | 9.32E+01 | 1.60150169 | 0.06297583891466016 | 2.8936 | 1.5329 | up |
| Lipid-Q-P-0511 | C46H82NO7P | PC(O-38:6) | GP | PC-O | LMGP01020064 | 1.40E+01 | 1.51E+01 | 6.15E+00 | 4.37E+01 | 1.53E+01 | 1.75E+01 | 1.83E+01 | 1.36E+01 | 2.25E+01 | 2.15E+01 | 1.83E+01 | 1.93E+01 | 1.91E+01 | 1.59E+01 | 1.32E+02 | 1.09E+02 | 1.71E+02 | 3.75E+01 | 1.54E+02 | 1.90E+01 | 1.58583261 | 0.03434442956001749 | 3.7049 | 1.8894 | up |
| Lipid-Q-P-0826 | C47H86O6 | TG(44:2)_16:1 | GL | TG | LMGL03014140 | 4.03E+01 | 3.80E+01 | 1.29E+02 | 8.46E-01 | 2.16E+00 | 2.10E+00 | 2.29E+00 | 3.51E+00 | 1.22E+00 | 3.32E+00 | 9.92E-01 | 1.15E+00 | 8.04E-01 | 1.63E+00 | 5.45E+00 | 4.98E-01 | 2.48E+00 | 7.66E-01 | 8.43E-01 | 1.50E+00 | 1.57169429 | 0.1419596547228136 | 0.0722 | -3.7919 | down |
| Lipid-Q-P-0440 | C46H88NO8P | PC(38:2) | GP | PC | LMGP01010788 | 3.53E+00 | 3.96E+00 | 2.04E+00 | 4.19E+00 | 2.80E+00 | 3.36E+00 | 2.61E+00 | 2.05E+00 | 4.56E+00 | 4.07E+00 | 4.47E+00 | 7.47E+00 | 5.51E+00 | 1.93E+00 | 8.43E+00 | 6.28E+00 | 8.74E+00 | 7.60E+00 | 1.62E+01 | 2.96E+00 | 1.56657233 | 0.017855883664030042 | 2.097 | 1.0683 | up |
| Lipid-Q-P-0747 | C56H108O6 | TG(53:0)_18:0 | GL | TG | LMGL03015132 | 3.40E+00 | N/A | N/A | 5.63E-01 | 3.13E-01 | 2.06E-01 | 2.03E-01 | 2.51E-01 | 9.02E-02 | 3.23E-01 | 5.34E+00 | 2.82E+00 | 3.22E+00 | 2.42E-01 | 5.43E+00 | 7.80E+00 | 9.74E+00 | 1.95E+00 | 7.31E-01 | 2.68E-01 | 1.55382725 | 0.016706747321984884 | 5.6159 | 2.4895 | up |
| Lipid-Q-P-0680 | C45H93N2O6P | SM(d40:0) | SL | SM | LMSP03010022 | 1.12E+01 | 1.72E+01 | 4.22E+00 | 8.35E+00 | 1.14E+01 | 1.06E+01 | 1.28E+01 | 7.20E+00 | 1.76E+01 | 3.80E+00 | 2.34E+00 | 2.50E+00 | 2.40E+00 | 3.50E+00 | 7.47E+00 | 5.18E+00 | 6.37E+00 | 3.67E+00 | 4.91E+00 | 3.76E+00 | 1.52171647 | 0.002290732489025119 | 0.4034 | -1.3097 | down |
| Lipid-Q-P-0377 | C27H50NO7P | LPE(22:3/0:0) | GP | LPE | - | 1.78E+01 | 2.05E+01 | 3.08E+01 | 5.86E+00 | 7.64E+00 | 4.90E+00 | 3.14E+00 | 3.35E+00 | 3.87E+00 | 2.86E+00 | 1.79E+00 | 2.13E+00 | 1.55E+00 | 2.64E+00 | 6.23E+00 | 4.36E+00 | 4.01E+00 | 2.20E+00 | 3.75E+00 | 2.42E+00 | 1.51295614 | 0.04863284649067702 | 0.3084 | -1.6971 | down |
| Lipid-Q-N-0109 | C27H51O12P | LPI(18:1) | GP | LPI | LMGP06050005 | 1.08E+02 | 1.67E+02 | 1.73E+02 | 1.13E+02 | 2.16E+02 | 2.77E+02 | 2.36E+02 | 1.16E+02 | 3.26E+02 | 1.09E+02 | 1.06E+02 | 8.17E+01 | 8.82E+01 | 2.94E+01 | 8.82E+01 | 8.47E+01 | 8.17E+01 | 1.09E+02 | 1.38E+02 | 8.35E+01 | 1.5126661 | 0.0037019852719748403 | 0.4837 | -1.0478 | down |
| Lipid-Q-P-0060 | C45H72O2 | CE(18:4) | ST | CE | - | N/A | N/A | N/A | 8.71E+00 | N/A | N/A | N/A | N/A | N/A | N/A | 7.74E+01 | 7.87E+01 | 5.23E+01 | N/A | 3.36E+01 | 7.97E+01 | N/A | N/A | 3.63E+01 | N/A | 1.50796212 | N/A | 6.8478 | 2.7756 | up |
| Lipid-Q-P-0748 | C56H108O6 | TG(53:0)_17:0 | GL | TG | LMGL03010098 | 1.97E+00 | N/A | N/A | 3.25E-01 | N/A | N/A | N/A | N/A | 8.51E-02 | 5.48E-01 | 3.53E+00 | 1.37E+00 | 1.97E+00 | N/A | 3.08E+00 | 4.41E+00 | 4.72E+00 | 1.18E+00 | 3.39E-01 | N/A | 1.4878736 | 0.026320148610780827 | 3.5189 | 1.8151 | up |
| Lipid-Q-P-0768 | C45H84O6 | TG(42:1)_18:1 | GL | TG | - | 2.68E+01 | 2.98E+01 | 8.06E+01 | 8.62E-01 | 1.33E+00 | 9.56E-01 | 9.57E-01 | 9.48E-01 | 6.21E-01 | 2.26E+00 | 4.81E-01 | 4.53E-01 | 3.44E-01 | 5.36E-01 | 6.57E+00 | 2.51E-01 | 3.27E+00 | 1.92E-01 | 4.47E-02 | 3.47E-01 | 1.46445177 | 0.13936895137170058 | 0.086 | -3.5395 | down |
| Lipid-Q-P-0456 | C47H86NO8P | PC(39:4) | GP | PC | LMGP01011748 | 3.69E-01 | 7.91E-01 | 1.58E-01 | 1.07E+00 | 4.30E-01 | 4.11E-01 | 5.48E-01 | 3.18E-01 | 7.90E-01 | 5.68E-01 | 6.48E-01 | 7.87E-01 | 8.05E-01 | 4.58E-01 | 9.77E-01 | 1.52E+00 | 1.37E+00 | 4.02E-01 | 9.21E+00 | 8.84E-01 | 1.42695975 | 0.20248362325571015 | 3.1272 | 1.6449 | up |
| Lipid-Q-P-0199 | C43H80O5 | DG(18:1_22:1) | GL | DG | - | N/A | 1.05E-01 | 9.86E-01 | 1.35E+00 | N/A | N/A | N/A | N/A | N/A | N/A | 7.88E+00 | 5.53E+00 | 4.28E+00 | N/A | 7.35E+00 | 4.71E+00 | 2.96E+00 | 6.01E+00 | 7.80E-01 | N/A | 1.42336116 | 0.0013759138728710028 | 6.07 | 2.6017 | up |
| Lipid-Q-P-0772 | C47H88O6 | TG(44:1)_16:1 | GL | TG | LMGL03013322 | 6.53E+01 | 6.41E+01 | 1.95E+02 | 2.01E+00 | 2.48E+00 | 4.44E+00 | 3.69E+00 | 3.83E+00 | 3.12E+00 | 4.24E+00 | 5.38E+00 | 2.39E+00 | 4.01E+00 | 2.42E+00 | 8.68E+00 | 7.31E-01 | 3.84E+00 | 3.49E+00 | 1.07E+00 | 2.59E+00 | 1.42063326 | 0.14305125951181652 | 0.0994 | -3.3306 | down |
| Lipid-Q-N-0074 | C22H44O2 | FFA(22:0) | FA | FFA | LMFA01010022 | 4.90E+01 | 8.47E+01 | 1.08E+02 | 4.76E+01 | 3.68E+01 | 4.44E+01 | 4.41E+01 | 4.05E+01 | 3.30E+01 | 4.54E+01 | 1.20E+02 | 1.09E+02 | 1.17E+02 | 4.14E+01 | 1.58E+02 | 1.29E+02 | 1.35E+02 | 1.43E+02 | 8.50E+01 | 5.10E+01 | 1.42010347 | 0.0014836185657702069 | 2.0405 | 1.0289 | up |
| Lipid-Q-P-0353 | C21H44NO7P | LPE(0:0/16:0) | GP | LPE | LMGP02050036 | 1.10E+02 | 1.33E+02 | 5.89E+01 | 5.50E+01 | 3.47E+01 | 5.42E+01 | 6.05E+01 | 4.50E+01 | 2.72E+01 | 1.85E+01 | 4.11E+01 | 1.71E+01 | 3.25E+01 | 1.92E+01 | 1.85E+01 | 2.63E+01 | 4.42E+01 | 3.50E+01 | 2.73E+01 | 1.53E+01 | 1.40758984 | 0.020853374397957664 | 0.4627 | -1.1119 | down |
| Lipid-Q-P-0753 | C58H112O6 | TG(55:0)_16:0 | GL | TG | - | N/A | 1.34E-01 | 1.75E-01 | 8.23E-02 | N/A | N/A | N/A | N/A | N/A | N/A | 8.98E-01 | 7.62E-01 | 5.44E-01 | N/A | 1.60E+00 | 3.14E+00 | 2.96E+00 | 7.63E-01 | 3.86E-02 | N/A | 1.40199274 | 0.02015377162753641 | 10.2558 | 3.3584 | up |
| Lipid-Q-P-0105 | C34H67NO3 | Cer(d16:1/18:0) | SL | Cer | LMSP02010046 | 2.73E-01 | 9.18E-01 | 3.86E-01 | 7.68E-01 | 1.84E+00 | 2.20E+00 | 1.90E+00 | 2.01E+00 | 1.70E+00 | 1.10E+00 | 3.37E-01 | 5.03E-01 | 4.04E-01 | 1.23E-01 | 1.61E-01 | 1.25E-01 | 1.93E-01 | 2.48E-01 | 1.28E+00 | 4.16E-01 | 1.40179012 | 0.002444287055269752 | 0.2893 | -1.7894 | down |
| Lipid-Q-N-0095 | C22H34O2 | FFA(22:5) | FA | FFA | LMFA01030182 | 8.15E+00 | 3.07E+01 | 3.57E+01 | 1.95E+02 | 8.37E+01 | 1.15E+02 | 1.12E+02 | 5.38E+01 | 1.19E+02 | 4.42E+01 | 2.27E+02 | 1.87E+02 | 2.43E+02 | 3.14E+01 | 1.79E+02 | 1.19E+02 | 1.30E+02 | 1.48E+02 | 2.79E+02 | 8.64E+01 | 1.40015313 | 0.012463868695167237 | 2.0459 | 1.0327 | up |
| Lipid-Q-P-0774 | C47H88O6 | TG(44:1)_14:0 | GL | TG | LMGL03013217 | 1.08E+02 | 1.04E+02 | 2.98E+02 | 1.70E+00 | 1.87E+00 | 3.57E+00 | 4.07E+00 | 4.53E+00 | 2.16E+00 | 3.45E+00 | 3.29E+00 | 1.99E+00 | 3.29E+00 | 2.43E+00 | 1.25E+01 | 6.25E-01 | 4.96E+00 | 1.31E+00 | 1.13E+00 | 2.59E+00 | 1.39877266 | 0.13621933881902987 | 0.0642 | -3.9613 | down |
| Lipid-Q-N-0087 | C22H40O2 | FFA(22:2) | FA | FFA | LMFA01030131 | 1.39E+00 | 2.98E+00 | 4.18E+00 | 2.93E+01 | 1.04E+01 | 2.07E+01 | 3.38E+01 | 6.09E+00 | 1.33E+01 | 5.33E+00 | 2.08E+01 | 2.28E+01 | 2.59E+01 | 3.63E+00 | 6.18E+01 | 3.24E+01 | 4.14E+01 | 5.14E+01 | 2.73E+01 | 1.04E+01 | 1.39355986 | 0.0219972267812149 | 2.3376 | 1.225 | up |
| Lipid-Q-P-0827 | C47H86O6 | TG(44:2)_16:0 | GL | TG | - | 1.26E+02 | 1.68E+02 | 4.44E+02 | 3.91E+00 | 2.92E+00 | 3.34E+00 | 4.01E+00 | 2.36E+00 | 1.37E+00 | 2.16E+00 | 2.23E+00 | 8.98E-01 | 1.07E+00 | 5.86E-01 | 2.95E+01 | 4.41E-01 | 1.24E+01 | 1.43E+00 | 2.93E-01 | 6.76E-01 | 1.38420486 | 0.15202087604389777 | 0.0653 | -3.9368 | down |
| Lipid-Q-P-0035 | C45H80O2 | CE(18:0) | ST | CE | LMST01020007 | 1.24E+01 | 7.04E+00 | 2.06E+01 | 2.61E+02 | 4.70E+00 | 4.26E+00 | 4.33E+00 | 6.85E+00 | 3.21E+00 | N/A | 2.70E+03 | 1.69E+03 | 2.21E+03 | 6.16E+00 | 2.95E+03 | 1.58E+03 | 2.72E+03 | 2.04E+03 | 5.70E+02 | 2.13E+01 | 1.38219471 | 0.0012224162024926089 | 45.7161 | 5.5146 | up |
| Lipid-Q-N-0089 | C20H34O2 | FFA(20:3) | FA | FFA | LMFA01030157 | 1.42E+00 | 5.77E+00 | 5.94E+00 | 1.22E+02 | 4.05E+01 | 5.62E+01 | 5.48E+01 | 1.53E+01 | 3.38E+01 | 1.40E+01 | 4.19E+01 | 3.53E+01 | 4.69E+01 | 1.14E+01 | 1.20E+02 | 1.02E+02 | 1.08E+02 | 7.50E+01 | 2.55E+02 | 1.72E+01 | 1.37885873 | 0.09237768776635873 | 2.3275 | 1.2188 | up |
| Lipid-Q-P-0315 | C25H50NO7P | LPC(17:1/0:0) | GP | LPC | LMGP01050002 | 4.08E-01 | N/A | N/A | 8.71E-01 | 3.37E-01 | 3.57E-01 | 3.93E-01 | 3.68E-01 | 1.01E+00 | 3.01E-01 | N/A | N/A | N/A | N/A | N/A | N/A | N/A | N/A | N/A | 2.03E-01 | 1.37231972 | N/A | 0.4015 | -1.3165 | down |
| Lipid-Q-N-0034 | C20H32O3 | (±)12-HETE | FA | Eicosanoid | - | 5.17E+00 | 5.09E+00 | 1.70E+00 | 1.55E+00 | 8.67E-01 | 1.01E+00 | 6.49E-01 | 4.19E-01 | 2.11E-01 | 1.28E-01 | 9.20E-03 | 1.45E-01 | 1.20E-01 | 6.36E-01 | 9.10E-01 | 3.17E-01 | 3.07E-01 | 2.32E-01 | 2.98E-01 | 1.76E-01 | 1.36800236 | 0.049058814311530786 | 0.1877 | -2.4135 | down |
| Lipid-Q-P-0902 | C49H88O6 | TG(46:3)_18:1 | GL | TG | - | 1.60E+02 | 1.76E+02 | 4.73E+02 | 2.18E+00 | 2.47E+00 | 2.30E+00 | 2.44E+00 | 1.93E+00 | 3.97E-01 | 3.38E+00 | 2.10E+00 | 5.95E-01 | 9.45E-01 | 5.19E-01 | 1.52E+01 | 3.92E-01 | 7.06E+00 | 9.56E-01 | 2.37E-01 | 2.08E-01 | 1.36385811 | 0.1360694531494935 | 0.0343 | -4.8656 | down |
| Lipid-Q-P-0750 | C57H110O6 | TG(54:0)_18:0 | GL | TG | LMGL03010002 | 2.46E+01 | 4.57E+00 | 1.13E+01 | 7.00E+00 | 4.02E+00 | 3.70E+00 | 4.32E+00 | 5.32E+00 | 3.14E+00 | 6.94E+00 | 3.34E+01 | 2.07E+01 | 2.27E+01 | 4.07E+00 | 3.42E+01 | 4.01E+01 | 5.40E+01 | 1.96E+01 | 7.18E+00 | 4.30E+00 | 1.34901341 | 0.012456443800799065 | 3.2058 | 1.6807 | up |
| Lipid-Q-P-0334 | C30H54NO7P | LPC(22:4/0:0) | GP | LPC | LMGP01050124 | 1.57E+01 | 1.28E+01 | 9.67E+00 | 5.18E+00 | 1.90E+00 | 1.88E+00 | 1.82E+00 | 3.20E+00 | 3.74E+00 | 3.62E+00 | 1.97E+01 | 7.67E+00 | 1.30E+01 | 3.15E+00 | 1.52E+01 | 1.43E+01 | 2.29E+01 | 1.68E+01 | 4.09E+01 | 6.85E+00 | 1.34223424 | 0.01752694536862932 | 2.6978 | 1.4318 | up |
| Lipid-Q-P-0333 | C30H54NO7P | LPC(0:0/22:4) | GP | LPC | - | 1.47E+01 | 1.22E+01 | 9.97E+00 | 5.29E+00 | 1.84E+00 | 1.89E+00 | 1.95E+00 | 2.71E+00 | 3.44E+00 | 3.76E+00 | 1.89E+01 | 7.67E+00 | 1.30E+01 | 2.95E+00 | 1.44E+01 | 1.32E+01 | 2.35E+01 | 1.68E+01 | 3.72E+01 | 6.80E+00 | 1.34170338 | 0.01448418786726231 | 2.6736 | 1.4188 | up |
| Lipid-Q-P-0773 | C47H88O6 | TG(44:1)_10:0 | GL | TG | - | 9.36E+01 | 1.24E+02 | 2.49E+02 | 5.26E+00 | 2.85E+00 | 2.82E+00 | 3.33E+00 | 1.69E+00 | 9.77E-01 | 4.06E+00 | 3.20E+00 | 2.00E+00 | 2.47E+00 | 6.05E-01 | 1.49E+01 | 3.63E-01 | 9.17E+00 | 1.07E+00 | 1.91E-01 | 4.97E-01 | 1.33922246 | 0.12005445408596688 | 0.0708 | -3.8201 | down |
| Lipid-Q-N-0080 | C17H32O2 | FFA(17:1) | FA | FFA | LMFA01030059 | 2.37E+00 | 3.74E+00 | 3.95E+00 | 5.50E+00 | 3.47E+00 | 4.44E+00 | 4.02E+00 | 1.55E+00 | 4.46E+00 | 1.54E+00 | 8.59E+00 | 7.07E+00 | 9.88E+00 | 1.70E+00 | 1.56E+01 | 9.92E+00 | 1.19E+01 | 1.05E+01 | 1.23E+01 | 1.76E+00 | 1.33799884 | 0.003653869862332145 | 2.5444 | 1.3473 | up |
| Lipid-Q-P-0746 | C55H106O6 | TG(52:0)_18:0 | GL | TG | LMGL03010069 | 1.75E+02 | 5.41E+01 | 7.44E+01 | 9.80E+01 | 2.20E+01 | 2.06E+01 | 2.46E+01 | 2.89E+01 | 1.84E+01 | 2.72E+01 | 3.35E+02 | 2.05E+02 | 2.32E+02 | 2.97E+01 | 1.47E+02 | 1.70E+02 | 2.23E+02 | 1.38E+02 | 4.18E+01 | 4.37E+01 | 1.33725459 | 0.011363457690627042 | 2.8814 | 1.5268 | up |
| Lipid-Q-P-0374 | C23H42NO7P | LPE(18:3/0:0) | GP | LPE | LMGP02050042 | 3.09E+01 | 1.43E+01 | 3.83E+01 | 5.65E+00 | 2.24E+00 | 2.71E+00 | 2.78E+00 | 3.81E+00 | 5.12E+00 | 1.40E+00 | 2.44E+00 | 3.57E+00 | 1.97E+00 | 2.75E+00 | 3.37E+00 | 3.71E+00 | 2.27E+00 | 2.99E+00 | 1.55E+00 | 6.17E-01 | 1.3290347 | 0.08175202141868748 | 0.2352 | -2.088 | down |
| Lipid-Q-P-0744 | C55H106O6 | TG(52:0)_20:0 | GL | TG | LMGL03014348 | 6.35E+01 | 2.01E+01 | 4.81E+01 | 2.74E+01 | 1.82E+00 | 2.10E+00 | 2.12E+00 | 3.48E+00 | 2.03E+00 | 2.22E+00 | 1.91E+02 | 9.40E+01 | 1.13E+02 | 2.63E+00 | 1.09E+02 | 1.57E+02 | 1.63E+02 | 1.13E+02 | 1.11E+01 | 1.05E+01 | 1.31617898 | 0.0049365879196938516 | 5.5845 | 2.4814 | up |
| Lipid-Q-P-0755 | C59H114O6 | TG(56:0)_18:0 | GL | TG | LMGL03010459 | 5.10E+00 | 5.74E-01 | 2.07E+00 | 6.08E-01 | 2.10E-01 | 2.75E-01 | 3.55E-01 | 3.33E-01 | 2.13E-01 | 4.95E-01 | 1.10E+01 | 4.09E+00 | 6.53E+00 | 2.06E-01 | 1.68E+01 | 1.99E+01 | 2.73E+01 | 7.14E+00 | 5.94E-01 | 3.21E-01 | 1.30823578 | 0.019194642828339475 | 9.1769 | 3.198 | up |
| Lipid-Q-P-0298 | C23H48NO7P | LPC(15:0/0:0) | GP | LPC | LMGP01050016 | 1.65E+00 | 8.32E-01 | 1.08E+00 | 8.92E-01 | 4.23E-01 | 4.05E-01 | 4.81E-01 | 6.49E-01 | 6.85E-01 | 5.23E-01 | 2.21E+00 | 8.76E-01 | 1.72E+00 | 5.80E-01 | 1.13E+00 | 1.46E+00 | 1.93E+00 | 1.50E+00 | 4.12E+00 | 5.93E-01 | 1.30249623 | 0.03254469209539311 | 2.115 | 1.0807 | up |
| Lipid-Q-P-0297 | C23H48NO7P | LPC(0:0/15:0) | GP | LPC | - | 1.55E+00 | 8.06E-01 | 1.03E+00 | 8.87E-01 | 4.19E-01 | 3.85E-01 | 4.08E-01 | 6.19E-01 | 6.47E-01 | 5.18E-01 | 1.98E+00 | 8.42E-01 | 1.87E+00 | 4.90E-01 | 1.15E+00 | 1.43E+00 | 1.80E+00 | 1.34E+00 | 3.98E+00 | 5.59E-01 | 1.30085868 | 0.033368230235507376 | 2.1261 | 1.0882 | up |
| Lipid-Q-P-0901 | C49H88O6 | TG(46:3)_18:2 | GL | TG | LMGL03013352 | 2.10E+02 | 2.40E+02 | 7.16E+02 | 1.57E+00 | 3.07E+00 | 2.15E+00 | 4.22E+00 | 2.65E+00 | 7.61E-01 | 2.67E+00 | 3.33E+00 | 1.48E+00 | 2.32E+00 | 7.30E-01 | 2.08E+01 | 3.35E-01 | 9.40E+00 | 2.08E+00 | 6.02E-01 | 5.90E-01 | 1.29989151 | 0.15040258556010255 | 0.0352 | -4.8283 | down |
| Lipid-Q-P-0296 | C22H46NO7P | LPC(14:0/0:0) | GP | LPC | LMGP01050012 | 4.03E+00 | 1.58E+00 | 2.05E+00 | 1.21E+00 | 4.41E-01 | 5.71E-01 | 5.24E-01 | 1.29E+00 | 1.50E+00 | 7.84E-01 | 4.13E+00 | 2.08E+00 | 2.94E+00 | 1.41E+00 | 2.96E+00 | 3.71E+00 | 4.54E+00 | 2.85E+00 | 5.00E+00 | 6.99E-01 | 1.29150588 | 0.008436615475610105 | 2.1672 | 1.1158 | up |
| Lipid-Q-P-0214 | C43H78O5 | DG(18:2_22:1) | GL | DG | LMGL02010180 | N/A | N/A | 1.49E+00 | 6.22E-01 | N/A | N/A | N/A | N/A | N/A | N/A | 7.45E+00 | 4.13E+00 | 4.49E+00 | N/A | 4.48E+00 | 1.72E+00 | 1.93E+00 | 7.62E+00 | N/A | N/A | 1.28908583 | N/A | 4.302 | 2.105 | up |
| Lipid-Q-P-0548 | C47H88NO8P | PE(42:3) | GP | PE | - | 3.33E-01 | 5.66E-01 | N/A | 9.65E-01 | 7.35E-01 | 6.91E-01 | 7.50E-01 | 7.30E-01 | 6.08E-01 | 1.24E+00 | 6.78E-01 | 6.82E-01 | 7.86E-01 | 4.89E-01 | 5.22E+00 | 3.59E+00 | 5.50E+00 | 1.70E+00 | 1.36E+00 | 8.26E-01 | 1.28732083 | 0.05739958069359649 | 2.8342 | 1.5029 | up |
| Lipid-Q-P-0194 | C39H72O5 | DG(16:0_20:2) | GL | DG | LMGL02010055 | 1.94E-02 | 1.06E+00 | 1.30E+00 | 1.90E+01 | 3.65E+00 | 6.61E+00 | 4.57E+00 | 2.51E+00 | 2.00E+00 | 1.61E+00 | 3.22E+01 | 1.78E+01 | 1.76E+01 | 1.40E+00 | 1.05E+01 | 4.07E+00 | 5.25E+00 | 1.70E+01 | 7.70E+00 | 2.25E+00 | 1.27631068 | 0.054126744822785684 | 2.7388 | 1.4535 | up |
| Lipid-Q-N-0019 | C18H30O3 | 9-oxoODE | FA | Eicosanoid | - | 6.46E-02 | 9.74E-02 | N/A | 5.34E-02 | N/A | N/A | N/A | N/A | N/A | 1.89E-04 | N/A | 2.44E-01 | 2.76E-01 | 5.85E-01 | 8.15E-02 | 1.06E-01 | 1.03E-01 | 1.60E+00 | 6.11E-03 | N/A | 1.27169272 | 0.1290568425537509 | 6.9596 | 2.799 | up |
| Lipid-Q-P-0752 | C57H110O6 | TG(54:0)_16:0 | GL | TG | LMGL03010175 | 4.25E+01 | 8.36E+00 | 2.95E+01 | 1.06E+01 | 1.90E+00 | 2.06E+00 | 1.93E+00 | 2.25E+00 | 1.35E+00 | 3.09E+00 | 1.27E+02 | 5.60E+01 | 6.98E+01 | 1.86E+00 | 6.68E+01 | 7.53E+01 | 9.99E+01 | 8.25E+01 | 7.65E+00 | 3.02E+00 | 1.26890189 | 0.005711505837085626 | 5.7 | 2.511 | up |
| Lipid-Q-P-0314 | C24H48NO7P | LPC(16:1/0:0) | GP | LPC | LMGP01050021 | 4.58E+01 | 2.13E+01 | 3.14E+01 | 2.40E+01 | 7.84E+00 | 8.11E+00 | 9.75E+00 | 1.31E+01 | 2.66E+01 | 1.05E+01 | 8.39E+01 | 2.37E+01 | 5.47E+01 | 5.64E+00 | 4.98E+01 | 5.13E+01 | 7.25E+01 | 5.48E+01 | 9.11E+01 | 1.97E+01 | 1.2613482 | 0.007275287853751676 | 2.5554 | 1.3535 | up |
| Lipid-Q-N-0113 | C31H53O12P | LPI(22:4) | GP | LPI | LMGP06050013 | N/A | N/A | N/A | 9.80E+00 | 2.44E+00 | 5.81E+00 | N/A | N/A | N/A | N/A | 1.21E+01 | N/A | 1.32E+01 | N/A | 1.59E+01 | 1.94E+01 | 3.13E+01 | 9.52E+00 | 1.40E+01 | N/A | 1.24829121 | 0.01823820855862246 | 2.739 | 1.4536 | up |
| Lipid-Q-P-0283 | C48H91NO13 | ex2Cer(d18:1/18: | SL | HexCer | - | 5.67E-01 | 6.89E-01 | 7.05E-02 | 2.58E-01 | 3.32E-01 | 3.04E-01 | 2.94E-01 | N/A | N/A | 4.38E-01 | N/A | N/A | N/A | N/A | 1.96E-01 | N/A | N/A | 1.29E-01 | N/A | 1.48E-01 | 1.24678689 | 0.017402705610721287 | 0.4272 | -1.227 | down |
| Lipid-Q-P-0282 | C46H87NO13 | ex2Cer(d18:1/16: | SL | HexCer | - | 2.85E+01 | 3.19E+01 | 3.85E+00 | 2.37E+00 | 3.76E+00 | 4.60E+00 | 8.02E+00 | 4.36E+00 | 2.02E+00 | 6.74E+00 | 2.28E+00 | 5.13E+00 | 2.67E+00 | 3.17E+00 | 2.83E+00 | 2.47E+00 | 1.97E+00 | 3.94E+00 | 2.74E+00 | 4.01E+00 | 1.24468248 | 0.09608416881251065 | 0.3247 | -1.6228 | down |
| Lipid-Q-P-0823 | C45H82O6 | TG(42:2)_18:2 | GL | TG | - | 1.74E+01 | 2.27E+01 | 6.92E+01 | 8.82E-01 | 9.89E-01 | 9.75E-01 | 1.06E+00 | 8.20E-01 | 2.44E-01 | 1.43E+00 | 2.44E-01 | 1.97E-01 | 2.16E-01 | 2.11E-01 | 6.20E+00 | 1.11E-01 | 3.01E+00 | 1.73E-01 | 7.21E-02 | N/A | 1.24445783 | 0.16641882469729177 | 0.1001 | -3.3205 | down |
| Lipid-Q-N-0079 | C16H30O2 | FFA(16:1) | FA | FFA | LMFA01030054 | 1.67E+01 | 3.04E+01 | 3.36E+01 | 4.42E+01 | 3.96E+01 | 5.14E+01 | 4.29E+01 | 1.70E+01 | 5.94E+01 | 1.17E+01 | 7.94E+01 | 6.08E+01 | 9.49E+01 | 1.29E+01 | 2.30E+02 | 1.08E+02 | 1.54E+02 | 1.19E+02 | 7.84E+01 | 2.08E+01 | 1.24435041 | 0.014437646240042125 | 2.7603 | 1.4648 | up |
| Lipid-Q-P-0197 | C41H76O5 | DG(18:1_20:1) | GL | DG | LMGL02010090 | 4.60E+00 | 7.57E+00 | 2.09E+01 | 2.14E+01 | 7.17E-01 | 1.97E+00 | 1.82E+00 | 3.91E-01 | 1.25E+00 | 1.20E+00 | 6.06E+01 | 2.69E+01 | 3.54E+01 | 1.21E+00 | 6.85E+01 | 2.49E+01 | 3.77E+01 | 2.82E+01 | 5.83E+00 | 1.14E+00 | 1.24434753 | 0.01281221199431726 | 4.7036 | 2.2338 | up |
| Lipid-Q-P-0179 | C39H74O5 | DG(18:0_18:1) | GL | DG | LMGL02010043 | 1.35E+01 | 3.46E+01 | 4.57E+01 | 4.46E+01 | 1.67E+01 | 2.99E+01 | 2.63E+01 | 1.86E+01 | 1.41E+01 | 1.82E+01 | 1.62E+02 | 8.25E+01 | 1.09E+02 | 1.02E+01 | 1.71E+02 | 8.70E+01 | 9.05E+01 | 1.01E+02 | 1.99E+01 | 1.49E+01 | 1.24411523 | 0.009758455843905767 | 3.2323 | 1.6926 | up |
| Lipid-Q-P-0332 | C28H50NO7P | LPC(20:4/0:0) | GP | LPC | LMGP01050048 | 2.59E+02 | 2.07E+02 | 1.69E+02 | 1.73E+02 | 6.61E+01 | 6.72E+01 | 7.11E+01 | 1.04E+02 | 1.50E+02 | 8.04E+01 | 3.16E+02 | 1.33E+02 | 2.58E+02 | 5.23E+01 | 2.76E+02 | 3.20E+02 | 5.07E+02 | 2.31E+02 | 1.17E+03 | 1.54E+02 | 1.23490528 | 0.07041548720969254 | 2.5339 | 1.3414 | up |
| Lipid-Q-P-0896 | C47H84O6 | TG(44:3)_18:2 | GL | TG | - | 4.74E+01 | 5.63E+01 | 1.59E+02 | 8.48E-01 | 1.72E+00 | 1.15E+00 | 1.44E+00 | 8.66E-01 | 4.22E-01 | 1.40E+00 | 5.76E-01 | 3.96E-01 | 3.80E-01 | 2.40E-01 | 1.15E+01 | 2.08E-01 | 5.24E+00 | 5.88E-01 | 3.91E-02 | N/A | 1.1910947 | 0.15690414939968275 | 0.0786 | -3.6693 | down |
| Lipid-Q-P-0895 | C47H84O6 | TG(44:3)_16:1 | GL | TG | LMGL03012816 | 1.43E+01 | 1.58E+01 | 4.52E+01 | 4.12E-01 | 5.20E-01 | 4.67E-01 | 7.22E-01 | 2.27E-01 | 2.14E-01 | 8.61E-01 | 3.14E-01 | 1.67E-01 | 1.60E-01 | N/A | 2.23E+00 | 8.06E-02 | 8.88E-01 | 6.76E-02 | 7.22E-02 | N/A | 1.18734171 | 0.1413568583629116 | 0.0632 | -3.9839 | down |
| Lipid-Q-P-0150 | C36H70O5 | DG(17:0_16:0) | GL | DG | - | N/A | N/A | 4.42E-01 | 1.09E+00 | 1.19E-01 | 2.77E-01 | 2.32E-01 | 1.14E-01 | 3.92E-01 | 3.54E-02 | 1.56E+00 | 8.18E-01 | 6.12E-01 | 1.87E-01 | 1.22E+00 | 3.00E-01 | 7.17E-01 | 7.38E-01 | 7.62E-01 | 2.81E-01 | 1.18542659 | 0.048951081470929705 | 2.1309 | 1.0915 | up |
| Lipid-Q-N-0088 | C18H30O2 | FFA(18:3) | FA | FFA | LMFA01030140 | 3.43E+00 | 5.51E+00 | 6.95E+00 | 1.10E+01 | 7.20E+00 | 7.35E+00 | 7.11E+00 | 2.77E+00 | 1.02E+01 | 2.48E+00 | 2.17E+01 | 1.54E+01 | 2.33E+01 | 2.61E+00 | 2.99E+01 | 1.11E+01 | 1.84E+01 | 3.58E+01 | 8.45E+00 | 3.40E+00 | 1.18497289 | 0.014149494400743088 | 2.656 | 1.4093 | up |
| Lipid-Q-P-0892 | C45H80O6 | TG(42:3)_16:1 | GL | TG | - | 1.63E+00 | 1.88E+00 | 4.93E+00 | N/A | 1.97E-01 | 2.52E-01 | N/A | N/A | N/A | 3.85E-01 | N/A | N/A | N/A | N/A | 5.66E-01 | N/A | 3.14E-01 | N/A | N/A | N/A | 1.18464358 | N/A | 0.2851 | -1.8105 | down |
| Lipid-Q-P-0738 | C53H102O6 | TG(50:0)_16:0 | GL | TG | LMGL03010004 | 9.63E+02 | 4.22E+02 | 5.82E+02 | 5.07E+02 | 8.23E+01 | 7.62E+01 | 9.02E+01 | 1.07E+02 | 7.90E+01 | 8.19E+01 | 2.05E+03 | 1.35E+03 | 1.57E+03 | 1.10E+02 | 8.67E+02 | 9.04E+02 | 1.21E+03 | 1.17E+03 | 1.63E+02 | 1.81E+02 | 1.18135496 | 0.012608956108808248 | .199600000000000 | 1.6779 | up |
| Lipid-Q-P-0762 | C63H122O6 | TG(60:0)_18:0 | GL | TG | LMGL03011114 | 1.73E-01 | N/A | 4.69E-02 | 8.08E-03 | 9.49E-03 | 1.57E-03 | 7.99E-03 | 2.28E-02 | N/A | N/A | 2.89E-01 | 9.37E-02 | 1.25E-01 | N/A | 4.56E-01 | 4.92E-01 | 6.20E-01 | 1.95E-01 | 4.92E-01 | 1.66E-02 | 1.17602015 | 0.005025876187098635 | 8.0112 | 3.002 | up |
| Lipid-Q-P-0177 | C38H72O5 | DG(16:0_19:1) | GL | DG | - | N/A | N/A | N/A | 4.95E-02 | N/A | N/A | N/A | N/A | N/A | N/A | 9.31E-01 | N/A | 1.69E-01 | N/A | 1.53E-01 | N/A | N/A | 7.05E-02 | 1.26E-02 | N/A | 1.17043731 | N/A | 5.3993 | 2.4328 | up |
| Lipid-Q-P-0723 | C45H86O6 | TG(42:0)_16:0 | GL | TG | LMGL03013211 | 2.92E+01 | 3.80E+01 | 9.34E+01 | 4.73E+00 | 5.11E+00 | 2.71E+00 | 4.37E+00 | 3.03E+00 | 1.57E+00 | 3.02E+00 | 4.13E+00 | 1.79E+00 | 2.06E+00 | 1.03E+00 | 6.43E+00 | 9.58E-01 | 4.01E+00 | 1.47E+00 | N/A | 1.36E+00 | 1.16263504 | 0.119294918705773 | 0.1394 | -2.8427 | down |
| Lipid-Q-P-0210 | C41H74O5 | DG(18:2_20:1) | GL | DG | LMGL02010099 | 2.64E+00 | 4.64E+00 | 1.46E+01 | 9.69E+00 | 7.46E-01 | 1.64E+00 | 1.52E+00 | 6.69E-01 | 9.03E-01 | 3.15E-01 | 5.04E+01 | 2.72E+01 | 2.84E+01 | 4.99E-01 | 4.20E+01 | 2.06E+01 | 2.30E+01 | 4.12E+01 | 3.10E+00 | 6.02E-01 | 1.16057554 | 0.006489654541371027 | 6.3404 | 2.6646 | up |
| Lipid-Q-P-0468 | C46H80NO8P | PC(38:6) | GP | PC | LMGP01010650 | 3.75E+01 | 1.83E+01 | 6.70E+00 | 4.27E+01 | 4.32E+01 | 4.90E+01 | 5.62E+01 | 2.95E+01 | 3.99E+01 | 2.08E+01 | 8.04E+00 | 1.36E+01 | 1.03E+01 | 1.72E+01 | 1.50E+01 | 1.14E+01 | 1.18E+01 | 9.66E+00 | 3.22E+01 | 2.26E+01 | 1.14903438 | 0.0033993721655917308 | 0.4414 | -1.1798 | down |
| Lipid-Q-P-0727 | C47H90O6 | TG(44:0)_16:0 | GL | TG | LMGL03012756 | 7.63E+01 | 8.66E+01 | 2.10E+02 | 9.59E+00 | 6.66E+00 | 8.08E+00 | 9.60E+00 | 9.31E+00 | 7.49E+00 | 1.24E+01 | 2.04E+01 | 8.90E+00 | 1.28E+01 | 7.71E+00 | 2.00E+01 | 4.48E+00 | 1.52E+01 | 1.14E+01 | 3.85E+00 | 5.32E+00 | 1.14839225 | 0.15262838063496367 | 0.2521 | -1.9879 | down |
| Lipid-Q-P-0835 | C49H90O6 | TG(46:2)_18:1 | GL | TG | LMGL03014145 | 2.74E+02 | 3.43E+02 | 8.04E+02 | 2.82E+00 | 4.01E+00 | 3.05E+00 | 3.45E+00 | 2.78E+00 | 1.77E+00 | 2.62E+00 | 6.81E+00 | 3.11E+00 | 5.78E+00 | 1.66E+00 | 3.72E+01 | 7.76E-01 | 1.44E+01 | 3.58E+00 | 1.05E+00 | 7.26E-01 | 1.13744574 | 0.13741267820831088 | 0.0521 | -4.2626 | down |
| Lipid-Q-P-1167 | C63H98O6 | TG(60:12)_22:6 | GL | TG | LMGL03012142 | 4.06E+00 | 6.29E+00 | 1.29E+01 | 1.92E+00 | 1.20E-01 | 2.20E-01 | 3.08E-01 | 3.07E-02 | 1.58E-01 | N/A | 5.59E-01 | 5.41E-01 | 3.86E-01 | 1.78E-01 | 1.19E-01 | N/A | N/A | 5.52E-03 | 9.69E-02 | 7.18E-02 | 1.13723669 | 0.10577384916157952 | 0.0846 | -3.5632 | down |
| Lipid-Q-P-0828 | C47H86O6 | TG(44:2)_14:0 | GL | TG | LMGL03013218 | 3.94E+01 | 4.06E+01 | 1.56E+02 | 1.10E-01 | 7.38E-01 | 3.64E-01 | 7.61E-01 | 1.24E+00 | 6.18E-01 | 5.44E-01 | 4.78E-01 | 2.61E-01 | 4.82E-01 | 5.13E-01 | 3.62E+00 | 2.21E-01 | 1.50E+00 | 5.64E-01 | 2.06E-01 | N/A | 1.13435949 | 0.17042902843260999 | 0.0363 | -4.7839 | down |
| Lipid-Q-N-0081 | C18H34O2 | FFA(18:1) | FA | FFA | LMFA01030061 | 1.23E+02 | 2.58E+02 | 3.92E+02 | 4.01E+02 | 3.43E+02 | 4.53E+02 | 4.15E+02 | 1.55E+02 | 3.88E+02 | 1.26E+02 | 6.08E+02 | 5.68E+02 | 7.24E+02 | 1.20E+02 | 1.20E+03 | 7.17E+02 | 9.56E+02 | 9.13E+02 | 4.44E+02 | 1.96E+02 | 1.13083845 | 0.01179872342596941 | 2.1118 | 1.0785 | up |
| Lipid-Q-P-1125 | C57H94O6 | TG(54:8)_18:2 | GL | TG | LMGL03010471 | 9.63E+01 | 7.52E+01 | 4.32E+02 | 1.70E+00 | 9.21E-01 | 7.68E-01 | 1.27E+00 | 1.11E+00 | 4.15E-01 | 3.98E-01 | 4.54E+00 | 2.51E+00 | 3.98E+00 | 1.30E+00 | 6.71E+00 | 3.50E-02 | 1.87E+00 | 2.39E+00 | 9.42E-02 | 1.45E-01 | 1.12890069 | 0.20324889296896761 | 0.0386 | -4.6953 | down |
| Lipid-Q-P-1155 | C61H98O6 | TG(58:10)_18:2 | GL | TG | LMGL03011316 | 8.98E+01 | 8.65E+01 | 3.01E+02 | 7.94E+00 | 1.39E+00 | 1.84E+00 | 2.18E+00 | 7.32E-01 | 1.15E+00 | 4.51E-01 | 1.84E+01 | 1.14E+01 | 1.58E+01 | 5.86E-01 | 2.82E+00 | N/A | 8.56E-01 | 2.90E+00 | 1.68E-01 | 3.22E-01 | 1.12131272 | 0.185113341571839 | 0.12 | -3.0589 | down |
| Lipid-Q-P-0532 | C38H72NO8P | PE(33:2) | GP | PE | LMGP02010522 | 2.85E-01 | 2.29E-01 | 2.02E-01 | 9.65E-01 | 1.59E+00 | 1.89E+00 | 1.84E+00 | 9.79E-01 | 1.08E+00 | 3.86E-01 | 3.02E-01 | 2.60E-01 | 2.49E-01 | 1.62E-01 | 3.01E-01 | 3.49E-01 | 2.77E-01 | 4.03E-01 | 3.07E-01 | 2.70E-01 | 1.11168972 | 0.012046351998963162 | 0.3051 | -1.7126 | down |
| Lipid-Q-P-0719 | C43H82O6 | TG(40:0)_16:0 | GL | TG | - | 3.64E+00 | 6.02E+00 | 1.48E+01 | 5.51E-01 | 7.71E-01 | 7.27E-01 | N/A | N/A | 6.12E-01 | N/A | N/A | N/A | 3.42E-01 | N/A | 2.36E+00 | N/A | 1.36E+00 | N/A | N/A | N/A | 1.10828651 | 0.2627279903166499 | 0.3494 | -1.517 | down |
| Lipid-Q-P-0549 | C39H70NO8P | PE(34:4) | GP | PE | LMGP02010935 | 2.21E-01 | 1.84E-01 | 1.34E-01 | 2.10E+00 | 4.00E+00 | 4.31E+00 | 4.90E+00 | 2.10E+00 | 3.61E+00 | 4.36E-01 | 1.82E-01 | 2.68E-01 | 1.64E-01 | 1.86E-01 | 3.13E-01 | 3.05E-01 | 2.49E-01 | 1.99E-01 | 5.24E-01 | 2.97E-01 | 1.10112812 | 0.01048628275860368 | 0.1222 | -3.0327 | down |
| Lipid-Q-P-0195 | C39H72O5 | DG(18:0_18:2) | GL | DG | LMGL02010050 | 1.98E+01 | 4.57E+01 | 1.08E+02 | 8.93E+01 | 4.85E+01 | 8.36E+01 | 7.66E+01 | 2.95E+01 | 2.76E+01 | 2.32E+01 | 3.84E+02 | 2.12E+02 | 2.62E+02 | 2.91E+01 | 2.12E+02 | 1.44E+02 | 1.49E+02 | 3.23E+02 | 2.62E+01 | 2.71E+01 | 1.08522526 | 0.014001777169954353 | 3.206 | 1.6808 | up |
| Lipid-Q-P-0223 | C43H76O5 | DG(18:0_22:4) | GL | DG | LMGL02010194 | 7.05E-01 | 1.65E+00 | 7.86E-01 | 5.86E+00 | 1.09E+00 | 1.47E+00 | 1.27E+00 | 1.55E+00 | 2.70E-01 | 2.39E+00 | 1.68E+01 | 1.15E+01 | 1.05E+01 | 1.89E+00 | 2.31E+00 | 7.47E-01 | 6.63E-01 | 3.26E+00 | 2.45E+00 | 1.53E+00 | 1.07739644 | 0.09046880832066709 | 3.0241 | 1.5965 | up |
| Lipid-Q-P-0278 | C48H89NO8 | lexCer(d18:2/24:1 | SL | HexCer | - | 7.46E-02 | N/A | N/A | 2.45E-01 | 8.23E-01 | 5.90E-01 | 7.74E-01 | 2.54E-01 | 7.18E-01 | 4.18E-01 | N/A | N/A | N/A | 3.86E-02 | 6.56E-02 | N/A | N/A | 2.55E-01 | N/A | N/A | 1.07641373 | 0.014473902364834678 | 0.2459 | -2.0239 | down |
| Lipid-Q-P-0467 | C45H78NO8P | PC(37:6) | GP | PC | - | 2.65E-01 | 1.30E-01 | 9.77E-02 | 6.12E-01 | 5.09E-01 | 6.48E-01 | 7.16E-01 | 3.15E-01 | 5.99E-01 | 2.32E-01 | 8.24E-02 | 1.46E-01 | 1.03E-01 | 1.99E-01 | 1.89E-01 | 1.01E-01 | 1.61E-01 | 1.12E-01 | 4.83E-01 | 1.54E-01 | 1.07144846 | 0.011129947591286302 | 0.4194 | -1.2536 | down |
| Lipid-Q-P-0765 | [illegible] | [illegible] | [illegible] | [illegible] | [illegible] | [illegible] | [illegible] | [illegible] | [illegible] | [illegible] | [illegible] | [illegible] | [illegible] | [illegible] | [illegible] | [illegible] | [illegible] | [illegible] | [illegible] | [illegible] | [illegible] | [illegible] | [illegible] | [illegible] | [illegible] | [illegible] | [illegible] | [illegible] | [illegible] | [illegible] |
| Lipid-Q-P-0171 | [illegible] | [illegible] | [illegible] | [illegible] | [illegible] | [illegible] | [illegible] | [illegible] | [illegible] | [illegible] | [illegible] | [illegible] | [illegible] | [illegible] | [illegible] | [illegible] | [illegible] | [illegible] | [illegible] | [illegible] | [illegible] | [illegible] | [illegible] | [illegible] | [illegible] | [illegible] | [illegible] | [illegible] | [illegible] | [illegible] |
| Lipid-Q-P-0230 | [illegible] | [illegible] | [illegible] | [illegible] | [illegible] | [illegible] | [illegible] | [illegible] | [illegible] | [illegible] | [illegible] | [illegible] | [illegible] | [illegible] | [illegible] | [illegible] | [illegible] | [illegible] | [illegible] | [illegible] | [illegible] | [illegible] | [illegible] | [illegible] | [illegible] | [illegible] | [illegible] | [illegible] | [illegible] | [illegible] |
| Lipid-Q-P-0363 | [illegible] | [illegible] | [illegible] | [illegible] | [illegible] | [illegible] | [illegible] | [illegible] | [illegible] | [illegible] | [illegible] | [illegible] | [illegible] | [illegible] | [illegible] | [illegible] | [illegible] | [illegible] | [illegible] | [illegible] | [illegible] | [illegible] | [illegible] | [illegible] | [illegible] | [illegible] | [illegible] | [illegible] | [illegible] | [illegible] |
| Lipid-Q-P-0114 | [illegible] | [illegible] | [illegible] | [illegible] | [illegible] | [illegible] | [illegible] | [illegible] | [illegible] | [illegible] | [illegible] | [illegible] | [illegible] | [illegible] | [illegible] | [illegible] | [illegible] | [illegible] | [illegible] | [illegible] | [illegible] | [illegible] | [illegible] | [illegible] | [illegible] | [illegible] | [illegible] | [illegible] | [illegible] | [illegible] |
| Lipid-Q-P-0211 | [illegible] | [illegible] | [illegible] | [illegible] | [illegible] | [illegible] | [illegible] | [illegible] | [illegible] | [illegible] | [illegible] | [illegible] | [illegible] | [illegible] | [illegible] | [illegible] | [illegible] | [illegible] | [illegible] | [illegible] | [illegible] | [illegible] | [illegible] | [illegible] | [illegible] | [illegible] | [illegible] | [illegible] | [illegible] | [illegible] |
| Lipid-Q-P-1023 | [illegible] | [illegible] | [illegible] | [illegible] | [illegible] | [illegible] | [illegible] | [illegible] | [illegible] | [illegible] | [illegible] | [illegible] | [illegible] | [illegible] | [illegible] | [illegible] | [illegible] | [illegible] | [illegible] | [illegible] | [illegible] | [illegible] | [illegible] | [illegible] | [illegible] | [illegible] | [illegible] | [illegible] | [illegible] | [illegible] |
| Lipid-Q-N-0118 | [illegible] | [illegible] | [illegible] | [illegible] | [illegible] | [illegible] | [illegible] | [illegible] | [illegible] | [illegible] | [illegible] | [illegible] | [illegible] | [illegible] | [illegible] | [illegible] | [illegible] | [illegible] | [illegible] | [illegible] | [illegible] | [illegible] | [illegible] | [illegible] | [illegible] | [illegible] | [illegible] | [illegible] | [illegible] | [illegible] |
